# Screening strategies for the early detection of non-cardia gastric cancer in low- to moderate-prevalence regions: Asia-Pacific gastroesophageal cancer congress consensus 2026

**DOI:** 10.1007/s10120-026-01800-4

**Published:** 2026-10-14

**Authors:** Ayaka Takasu, Fumiaki Ishibashi, Rapat Pittayanon, Hon Chi Yip, James Weiquan Li, Munkhsaruul Bayaraa, Hyunsoo Chung, Do Hoon Kim, Kamin Harinwan, Quang Dinh Le, Adolfo Parra-Blanco, Paulo Kassab, Vincenzo Giorgio Mirante, José Murillo-Rodriguez, Regina Rie Imada, Renata Nobre Moura, Sabina Beg, Pedro Genaro Delgado-Guillena, Marta Rodriguez-Carrasco, Seung-Hun Chon, Chul S. Hyun, Grigorios Christodoulidis, Vo Duy Long, Zili Xiao, Sergey Kashin, Roman Kuvaev, Ian Yu-Hong Wong, Robert Bechara, Mitsuru Esaki, Waku Hatta, Motohiko Kato, Seiichiro Abe, Sho Suzuki, Kohei Takizawa, Joo Ha Hwang, Wai Keung Leung, Duc Trong Quach, Matsuda Satoru, Han-Kwang Yang, Takuji Gotoda

**Affiliations:** 1 Department of Gastroenterology, Cancer Institute Hospital, Japanese Foundation for Cancer Research, Tokyo, Japan; 2 Division of Gastroenterology and Hepatology, Department of Medicine, Nihon University School of Medicine, Tokyo, Japan; 3 Digestive Disease Center, Showa Medical University Northern Yokohama Hospital, Kanagawa, 224-8503 Japan; 4 Division of Gastroenterology, Department of Medicine, Faculty of Medicine, Chulalongkorn University and King Chulalongkorn Memorial Hospital, Bangkok, Thailand; 5 Department of Surgery, Faculty of Medicine, The Chinese University of Hong Kong, Hong Kong SAR, China; 6 Division of Medicine and Inpatient Care, Sengkang General Hospital, Singapore, Singapore; 7 Department of Gastroenterology and Endoscopy, UB Songdo Hospital, Ulaanbaatar, Mongolia; 8 Department of Internal Medicine, Seoul National University College of Medicine, Seoul, Korea; 9 Department of Gastroenterology, Asan Medical Center, University of Ulsan College of Medicine, Seoul, Korea; 10 Division of Gastroenterology and Hepatology, Department of Medicine, Phramongkutklao Hospital and College of Medicine, Bangkok, Thailand; 11 Department of Internal Medicine, School of Medicine, University of Medicine and Pharmacy at Ho Chi Minh City, Ho Chi Minh, Vietnam; 12 Department of Gastroenterology, Nhan Dan Gia Dinh Hospital, Ho Chi Minh City, Vietnam; 13 NIHR Nottingham Digestive Diseases Biomedical Research Unit, Nottingham University Hospitals NHS Trust, Nottingham, United Kingdom of Great Britain and Northern Ireland; 14 Gastroesophageal and Bariatric Division, Department of Surgery, Hospital and Medical School, Santa Casa of São Paulo, São Paulo, Brazil; 15 Gastroenterology Unit A, Borgo Trento Hospital, Azienda Ospedaliera Universitaria Integrata Di Verona, Verona, Italy; 16 Department of Surgery, Hospital Mexico, Costa Rican Social Security, San José, Costa Rica; 17 Santa Casa de Misericórdia de São Paulo, São Paulo, Brazil; 18 Department of Gastroenterology, São Paulo Cancer Institute (ICESP), University of São Paulo School of Medicine, São Paulo, Brazil; 19 Department of Gastroenterology East and North Hertfordshire NHS Trust, Hertfordshire, UK; 20 Department of Gastroenterology, Hospital de Mérida, Badajoz, Spain; 21 RISE@CI-IPO (Health Research Network), Portuguese Oncology Institute of Porto (IPO Porto), Porto, Portugal; 22 Department of Gastroenterology and Hepatology, Faculty of Medicine and University Hospital of Cologne, University of Cologne, Cologne, Germany; 23 Section of Digestive Diseases, Yale School of Medicine, New Haven, CT USA; 24 Department of General Surgery, University Hospital of Larissa, Larissa, Greece; 25 Department of Gastrointestinal Surgery, University Medical Center, University of Medicine and Pharmacy at Ho Chi Minh City, Ho Chi Minh City, Vietnam; 26 Department of Gastrointestinal Endoscopy, Huadong Hospital, Fudan University, Shanghai, China; 27 Department of Endoscopy, Yaroslavl Regional Cancer Hospital, Yaroslavl State Medical University, Yaroslavl, Russia; 28 Department of Endoscopy, Yaroslavl Regional Cancer Hospital, Yaroslavl, Russia; 29 Department of Surgery, School of Clinical Medicine, LKS Faculty of Medicine, The University of Hong Kong, Hong Kong SAR, China; 30 Department of Medicine, Division of Gastroenterology, Kingston Health Sciences Centre, Kingston, ON Canada; 31 Department of Medicine and Bioregulatory Science, Graduate School of Medical Sciences, Kyushu University, Fukuoka, Japan; 32 Division of Gastroenterology, Tohoku University Graduate School of Medicine, Sendai, Miyagi Japan; 33 Center for Diagnostic and Therapeutic Endoscopy, Keio University School of Medicine, Tokyo, Japan; 34 Endoscopy Division, National Cancer Center Hospital, Tokyo, Japan; 35 Department of Gastroenterology, International University of Health and Welfare Ichikawa General Hospital, Ichikawa, Japan; 36 Department of Gastroenterology, Kanagawa Cancer Center, Kanagawa, Japan; 37 Division of Gastroenterology and Hepatology, Stanford University School of Medicine, Palo Alto, CA USA; 38 Department of Medicine, School of Clinical Medicine, Faculty of Medicine, Li Ka Shing, University of Hong Kong, Hong Kong, China; 39 Department of Surgery, Keio University School of Medicine, Tokyo, Japan; 40 National Cancer Center, Goyang, Korea

**Keywords:** Stomach neoplasms, Early detection of cancer, Risk assessment

## Abstract

**Background:**

Gastric cancer (GC) remains a major global health burden, and early detection is essential for improving patient outcomes. Although GC screening has been widely implemented in East Asian countries with a high disease burden, the optimal screening strategy for low- to moderate-prevalence regions remains unclear. To address this gap, the Asia Pacific Gastroesophageal Cancer Congress (APGCC) convened an international consensus meeting to develop evidence-based recommendations for improving the detection of non-cardia gastric cancer (NCGC).

**Methods:**

The APGCC working group developed 11 clinical questions (CQs) addressing GC screening, with a particular focus on NCGC. For each CQ, assigned authors conducted literature reviews using PubMed and prepared evidence summaries and draft recommendations. Consensus was achieved through a two-round modified Delphi process involving 37 international voting members.

**Results:**

The consensus recommendations addressed population-based screening, screening modalities, identification of high-risk individuals, quality indicators for esophagogastroduodenoscopy (EGD), image-enhanced endoscopy, and artificial intelligence-assisted endoscopy. Population-based endoscopic screening is not recommended in low- to moderate-prevalence regions because of insufficient evidence. However, if screening is performed, EGD is preferred over upper gastrointestinal series for the detection of early NCGC in asymptomatic individuals. The recommendations also emphasize the importance of high-quality endoscopic examinations, risk-stratified screening using serum biomarkers, and the appropriate integration of advanced endoscopic technologies.

**Conclusions:**

These APGCC consensus recommendations provide practical guidance for improving the early detection of NCGC in low- to moderate-prevalence regions. This consensus also highlights important evidence gaps and establishes a framework for future research and the development of region-specific GC screening strategies.

**Supplementary Information:**

The online version contains supplementary material available at https://doi.org/10.1007/s10120-026-01800-4.

## Introduction

Gastric cancer (GC) is the fifth leading cause of cancer-related death worldwide [1]. *Helicobacter pylori* (*H. pylori*), classified by the World Health Organization as a Group 1 carcinogen, is the principal etiological factor for GC [2]. Alongside primary prevention through *H. pylori* eradication, GC screening has long been an important public health strategy in countries and regions with a high prevalence of *H. pylori* infection and correspondingly high risk of GC. In particular, East Asian countries, including Japan, Korea, and China, had a very high prevalence of *H. pylori* infection until the 2000s and consequently experienced a higher burden of GC incidence and mortality [3]. As a result, extensive evidence has accumulated to support the effectiveness of GC screening from both policy and clinical perspectives.

Improvements in sanitation and living conditions have led to a marked decline in *H. pylori* infection, particularly among younger generations, even in these historically high-prevalence regions [4, 5]. Consequently, the population distribution of GC risk has changed substantially. Therefore, careful consideration is required before applying screening strategies within this evolving sociomedical landscape without appropriate modification. Furthermore, although the overall prevalence of GC remains relatively low in many countries and regions, increasingly diverse populations include individuals who were born in or originate from high-prevalence regions. This evolving epidemiological landscape underscores the importance of individualized risk assessment in addition to population-level prevalence. In many Asian countries outside East Asia, where the prevalence of *H. pylori* infection is relatively low and the risk of GC is considered low to moderate, the optimal approach to GC screening remains uncertain.

To address these issues, a consensus meeting on GC screening was convened in March 2026 in Japan during the 11th Asia Pacific Gastroesophageal Cancer Congress (APGCC) as part of a working group of the International Gastric Cancer Association. The primary objective of this meeting was to facilitate the standardization of GC screening approaches across low- to moderate-prevalence regions participating in the APGCC. Eleven clinical questions (CQs), developed by the core members of the working group, were evaluated through a Delphi process involving 37 international experts from Asia and other regions worldwide. Based on the outcomes of this consensus process, the present report provides recommendations regarding appropriate GC screening strategies for low- to moderate-prevalence regions.

In this consensus meeting, particular emphasis was placed on the importance of early GC detection. Therefore, the discussion focused on non-cardia gastric cancer (NCGC), excluding gastric cardia cancer and gastroesophageal junction cancer, which are more frequently diagnosed at advanced stages [6, 7].

## Methods

### Development of CQs

The consensus process was developed in accordance with the Grading of Recommendations Assessment, Development and Evaluation (GRADE) approach, with predefined modifications, and conducted using a modified Delphi process [8]. The core members of the working group developed 11 CQs addressing key issues in GC screening based on the PICO (Population, Intervention, Comparison, Outcome) framework. Each CQ was assigned to two or three CQ authors, who were responsible for reviewing the available evidence, preparing an evidence summary, and drafting the corresponding recommendation statement and explanatory notes.

### Definition of low- to moderate-prevalence GC regions

For the purposes of this consensus statement, geographic regions were stratified according to the age-standardized incidence rate (ASR; world standard population) of GC, expressed per 100,000 person-years, using the widely adopted three-tier classification of high-, intermediate-, and low-risk areas: high-risk, ASR ≥ 20; intermediate-risk, ASR 10–19.9; and low-risk, ASR < 10 [9, 10]. Regions classified as high-risk under this scheme (e.g., Japan, the Republic of Korea, Mongolia, and parts of China) were excluded from the scope of the present consensus. “Low- to moderate-prevalence regions” were therefore operationally defined as countries or regions with a combined male and female ASR for GC of < 20 per 100,000 person-years, corresponding to the intermediate- and low-risk strata of this classification. ASR estimates were derived from the most recent GLOBOCAN 2022 dataset [1].

### Literature review

For each CQ, two CQ authors independently conducted a structured literature search using MEDLINE (via PubMed) to identify relevant English-language publications. Predefined search strategies were used for each CQ, and the detailed search strategies and numbers of records identified are provided in Supplementary Table 1. The two authors independently reviewed the retrieved articles, selected the studies to be cited, and prepared draft recommendation statements and explanatory notes based on the available evidence. The certainty of the evidence supporting each recommendation was assessed according to the GRADE methodology. The draft recommendation statements, assigned levels of evidence, and explanatory notes were subsequently reviewed and refined by the core members of the APGCC consensus group. The revised drafts were then circulated to all voting members via a web-based platform for review and discussion before the first-round voting.

### Delphi consensus process

**Fig. 1:**
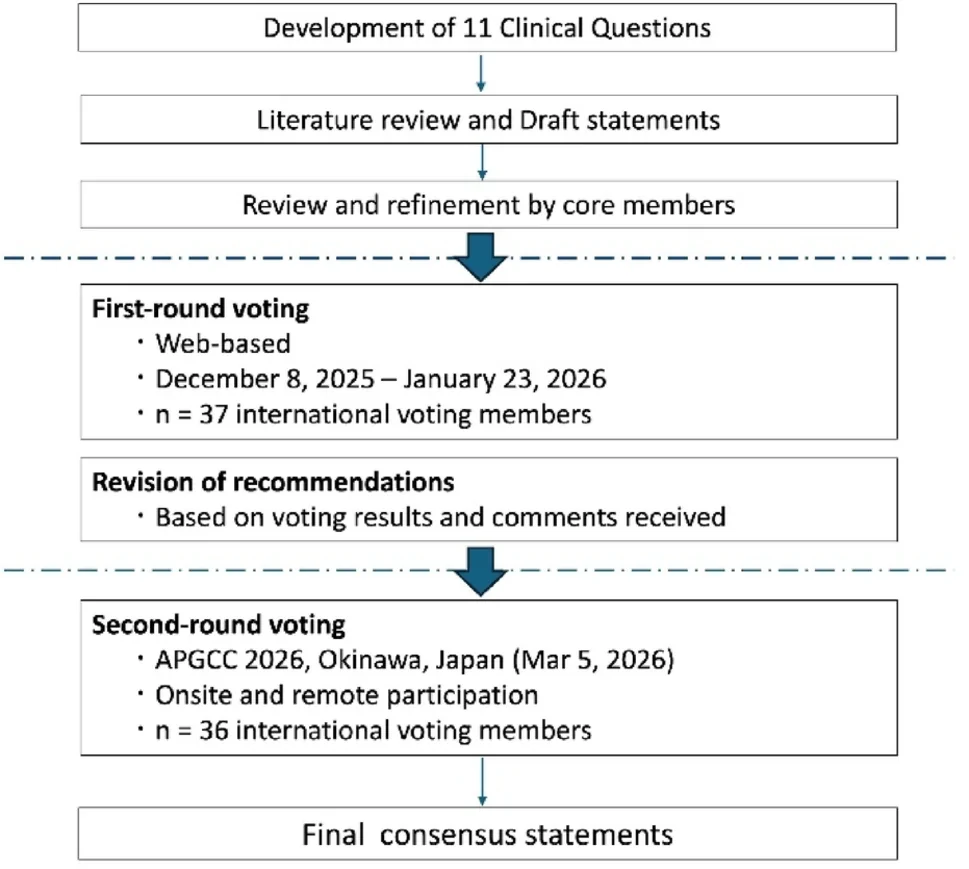
Development process of the APGCC consensus recommendations. The 11 clinical questions (CQs) were developed, followed by literature review, preparation and review of draft recommendations, and a two-round modified Delphi process. Draft recommendations were revised based on the first-round voting results and comments before the final consensus recommendations were established

Consensus was established using a two-round modified Delphi process involving 37 international voting members, including the CQ authors (Fig. 1). The first-round voting was conducted via a web-based platform between December 8, 2025, and January 23, 2026. Voting members evaluated each draft recommendation and were invited to provide comments regarding the wording and supporting evidence. Following the first-round voting, the CQ authors revised the recommendation statements and explanatory notes on the basis of the voting results and comments received. The second-round voting was conducted during the APGCC 2026 consensus meeting in Okinawa on March 5, 2026, with participation either onsite or remotely. In the second round of voting, 36 members participated because one member was absent. Each CQ was presented by the assigned CQ authors, followed by panel discussion and confidential electronic voting using the polling function of the virtual meeting platform. During the on-site voting, several members abstained or selected “Not graded” for specific CQs. Consequently, the number of voting participants varied across the CQs.

### Consensus definition

**Fig. 2:**
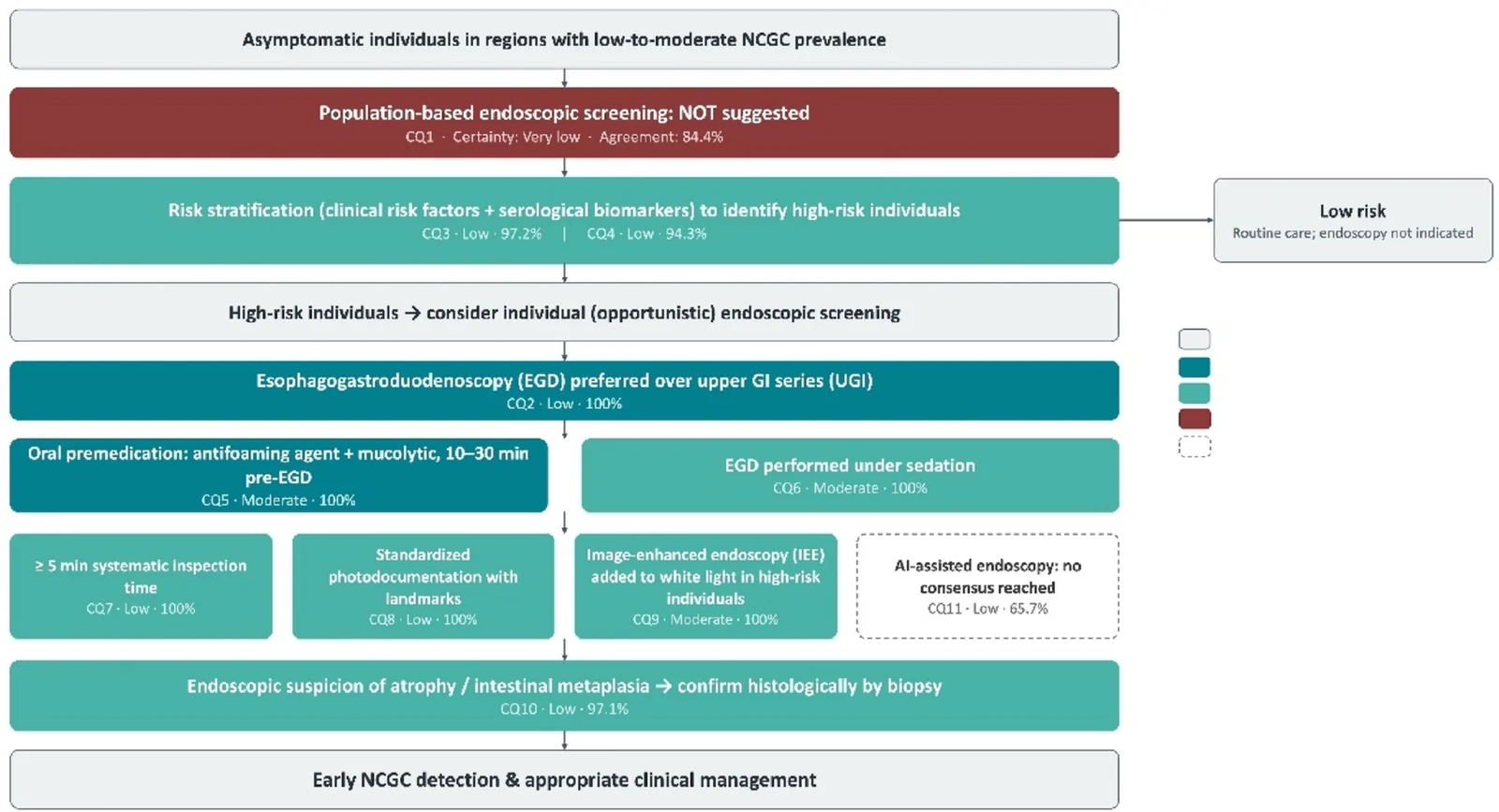
Recommended clinical pathway for early detection of non-cardia gastric cancer (NCGC) in low- to moderate-prevalence regions. Summary algorithm integrating the APGCC consensus recommendations (CQ1–CQ11). Green boxes indicate recommended practices, red boxes indicate interventions that are not recommended, gray boxes represent process or population steps, and dashed boxes indicate areas where consensus was not reached. Certainty of evidence was assessed using the GRADE approach. Percentages indicate the level of agreement among voting panel members. Abbreviations: NCGC, non-cardia gastric cancer; EGD, esophagogastroduodenoscopy; UGI, upper gastrointestinal series; IEE, image-enhanced endoscopy; AI, artificial intelligence; IM, intestinal metaplasia

Recommendation categories consisted of “Strongly agree with the statement,” “Agree with the statement,” “Disagree with the statement,” “Strongly disagree with the statement,” and “Not graded.” Consensus was predefined as at least 80% agreement (defined as “Strongly agree” or “Agree”) among voting members. For the wording of the final recommendations, statements for which ≥80% of voting members selected “Strongly agree” were expressed as “We recommend,” whereas statements that achieved the consensus threshold but had <80% “Strongly agree” were expressed as “We suggest.” Recommendation statements that achieved the predefined consensus threshold after the second-round voting were adopted as the final consensus recommendations. The finalized recommendation statements, levels of evidence, and final voting results for each CQ are summarized in Table 1, and the full voting results for each CQ are presented in Table 2. The overall recommended clinical pathway integrating the final APGCC consensus recommendations is summarized in Fig. 2.

**Table 1:** Clinical questions and statements

CQ	Clinical question	Statement	Certainty of evidence	Agreement, %
CQ1	Should a population-based endoscopy screening program be implemented for the detection of early NCGC among asymptomatic individuals in regions with low- to moderate-prevalence?	We do not suggest population-based endoscopy screening programs for the detection of early NCGC in asymptomatic individuals from regions with low- to moderate-prevalence	Very low	84.4
CQ2	Is EGD more effective than upper gastrointestinal series (UGI) for detecting early NCGC in asymptomatic individuals in regions with low- to moderate-prevalence?	We recommend EGD over UGI for the detection of early NCGC in asymptomatic individuals in regions with low- to moderate-prevalence	Low	100
CQ3	Is risk stratification beneficial for detecting early NCGC in regions with low- to moderate-prevalence?	We suggest a risk stratification strategy to identify high-risk individuals for endoscopic detection of early NCGC in regions with low- to moderate-prevalence	Low	97.2
CQ4	Can serological biomarkers assist in the identification of at-risk populations in low- to moderate-prevalence regions?	We suggest using serological biomarkers to identify individuals at high risk of NCGC in low- to moderate-prevalence regions	Low	94.3
CQ5	Should oral preparations be used routinely pre-EGD for detecting early NCGC?	We recommend routine oral premedication with an antifoaming agent and a mucolytic 10–30 min prior to diagnostic EGD for early NCGC detection to improve gastric mucosal visibility	Moderate	100
CQ6	Does EGD under sedation improve the detection rate of early NCGC?	We suggest EGD under sedation to improve the detection of early NCGC by facilitating optimal conditions for high-quality endoscopic assessment	Moderate	100
CQ7	What is the appropriate inspection time for EGD to improve the detection of early NCGC?	We suggest a total examination time of at least 5 min for systematic and comprehensive gastric mucosal inspection to improve the detection of early NCGC and ensure the quality of EGD	Low	100
CQ8	Should systematic photodocumentation with landmarks in EGD be standardized to improve the detection rate of early NCGC?	We suggest standardizing systematic photodocumentation with landmarks in EGD to improve the detection of early NCGC	Low	100
CQ9	Does IEE improve the detection of early NCGC?	We suggest the use of IEE, in addition to white-light endoscopy, for detecting early NCGC in high-risk individuals, although effectiveness varies across imaging modalities	Moderate	100
CQ10	Can endoscopic imaging accurately assess gastric atrophy or intestinal metaplasia to help identify high-risk patients for early NCGC?	We suggest confirming endoscopic suspicion of gastric atrophy and intestinal metaplasia histologically by biopsy in regions with a low- to moderate-prevalence	Low	97.1
CQ11	Should artificial intelligence (AI) endoscopy be used for improving the detection rate of early NCGC in low- to moderate-prevalence regions?	We could not reach consensus on whether to recommend or not recommend the routine use of AI-assisted endoscopy to improve the detection of early NCGC in low- to moderate-prevalence regions	Low	65.7%

CQ, clinical question; NCGC, non-cardia gastric cancer; EGD, esophagogastroduodenoscopy; IEE, image-enhanced endoscopy

**Table 2:** Final voting results for the recommendation statements

	Total number of votes	Strongly agree with the statement	Agree with the statement	Disagree with the statement	Strongly disagree with the statement	Not graded
CQ1	32	15 (46.9%)	12 (37.5%)	3 (9.4%)	1 (3.1%)	1 (3.1%)
CQ2	35	31 (88.6%)	4 (11.4%)	0 (0%)	0 (0%)	0 (0%)
CQ3	36	25 (69.4%)	10 (27.8%)	0 (0%)	0 (0%)	1 (2.8%)
CQ4	35	12 (34.3%)	21 (60.0%)	2 (5.7%)	0 (0%)	0 (0%)
CQ5	34	29 (85.3%)	5 (14.7%)	0 (0%)	0 (0%)	0 (0%)
CQ6	36	28 (77.8%)	8 (22.2%)	0 (0%)	0 (0%)	0 (0%)
CQ7	34	27 (79.4%)	7 (20.6%)	0 (0%)	0 (0%)	0 (0%)
CQ8	35	26 (74.3%)	9 (25.7%)	0 (0%)	0 (0%)	0 (0%)
CQ9	36	27 (75.0%)	9 (25.0%)	0 (0%)	0 (0%)	0 (0%)
CQ10	34	15 (44.1%)	18 (52.9%)	0 (0%)	0 (0%)	1 (2.9%)
CQ11	35	13 (37.1%)	10 (28.6%)	3 (8.6%)	1 (2.9%)	8 (22.9%)

CQ, clinical question

## Clinical question

### Clinical question 1

Should a population-based endoscopy screening program be implemented for the detection of early NCGC among asymptomatic individuals in regions with low- to moderate- prevalence?

#### Statement

We do not suggest population-based endoscopy screening programs for the detection of early NCGC in asymptomatic individuals from regions with low- to moderate- prevalence.

Certainty of evidence: Very low.

Result of voting: 84.4% (Strongly agree, 46.9%; Agree, 37.5%).

#### Explanatory note

There have been no randomized controlled trials (RCTs) investigating the effectiveness of population-based endoscopy screening programs in low- to moderate-prevalence regions. Nevertheless, several studies from Japan and Korea suggest that esophagogastroduodenoscopy (EGD) screening itself may contribute to the earlier detection of GC, particularly among asymptomatic individuals.

In Japan and Korea, where endoscopic screening for GC has already been widely implemented, most early-stage GC cases detected through screening are asymptomatic. A retrospective cohort study from Korea demonstrated that asymptomatic individuals were significantly more likely to have early-stage GC than symptomatic patients (74.2% vs. 25.9% in 1996 and 78.1% vs. 35.7% in 2001; *P* < 0.001) [11]. Similarly, a Japanese case–control study reported that 75% of asymptomatic GC cases were detected at an early stage, 84% had no lymph node metastasis, and 97% were amenable to curative resection [12].

A prospective cohort study in the United Kingdom, where endoscopic screening for GC among asymptomatic individuals is not routinely performed, found that 93% of patients with upper gastrointestinal cancers, including GC, presented with alarm symptoms at diagnosis after a median symptom duration of only 8 weeks [13]. These findings support the importance of detecting GC before the onset of alarm symptoms.

While these findings support the potential value of EGD as a screening modality, evidence for population-based endoscopic screening programs in low- to moderate-prevalence regions remains limited. In contrast, organized population-based GC screening programs have been implemented in high-prevalence countries, and robust evidence supports the association between EGD screening and early GC detection. In Korea, the National Cancer Screening Program (NCSP) provides EGD screening for adults aged 40 to 74 years, whereas Japan offers organized EGD screening for individuals aged ≥50 years.

A retrospective cohort study comparing 45,654 GC cases detected through the NCSP and opportunistic screening in Korea found a significantly higher proportion of early GC in the NCSP group (74.0% vs. 53.8%, *P* = 0.046) [14]. Likewise, a study of 18,414 NCSP participants demonstrated that individuals undergoing EGD screening were more likely to be diagnosed with localized GC than those who were not screened (adjusted odds ratio [OR], 2.10; 95% confidence interval [CI], 1.90–2.33) [15]. In Japan, a prospective cohort study involving 80,272 individuals also reported a significantly higher proportion of localized GC among participants undergoing organized EGD screening (75.0% vs. 54.1%, *P* < 0.001) [16]. These findings suggest that implementing population-based GC screening programs in high-prevalence regions facilitates earlier detection of GC; however, whether similar benefits would be observed in low- to moderate-prevalence regions remains unclear.

The primary goal of EGD screening is not only the early detection of GC but also the reduction of GC-related mortality. A nationwide Korean study including 16,584,283 individuals demonstrated a significant reduction in GC mortality among those who underwent EGD screening (OR, 0.53; 95% CI, 0.51–0.56) [17]. Similarly, a Japanese case–control study reported a significantly lower risk of GC mortality 36 months after EGD screening (OR, 0.695; 95% CI, 0.489–0.986) [18]. A meta-analysis of 10 East Asian studies further confirmed the association between EGD screening and reduced GC mortality (relative risk [RR], 0.60; 95% CI, 0.49–0.73) [19].

However, no comparable evidence is currently available for low- to moderate-prevalence regions outside East Asia. Taken together, although EGD screening may facilitate earlier detection of GC, current evidence remains insufficient to support population-based endoscopy screening programs for asymptomatic individuals in low- to moderate-prevalence regions. Further studies are needed to clarify the appropriate target population and optimal screening strategy in these regions. Importantly, this recommendation is not intended to discourage opportunistic EGD screening in higher-risk individuals.

### Clinical question 2

Is EGD more effective than upper gastrointestinal series (UGI) for detecting early NCGC in asymptomatic individuals in regions with low- to moderate-prevalence?

#### Statement

We recommend EGD over UGI for the detection of early NCGC in asymptomatic individuals in regions with low- to moderate-prevalence.

Certainty of evidence: Low.

Result of voting: 100% (Strongly agree, 88.6%; Agree, 11.4%).

#### Explanatory note

Currently, no RCTs have directly compared EGD and UGI using barium contrast for detecting early NCGC in asymptomatic individuals in regions with low- to moderate-prevalence. However, numerous retrospective and population-based studies comparing EGD and UGI for the detection of early GC have been reported from Japan and Korea, where GC prevalence is high and population-based screening programs using either EGD or UGI have been implemented.

In Korea and Japan, the high prevalence of GC is partly attributable to the high prevalence of *H. pylori* infection in the general population. In particular, in Korea, the prevalence was approximately 66.9% in 1998 and 59.6% in 2005 across all generations [20].

Given this background, the NCSP evaluated nationwide GC screening using either EGD or UGI based on data collected between 2002 and 2005. EGD demonstrated substantially higher diagnostic performance than UGI; the detection rate for GC was 2.61 vs. 0.68 cases per 1,000 screenings, sensitivity was 69.0% vs. 36.7%, and positive predictive value was 6.2% vs. 1.7%, respectively [21]. For localized cancers, EGD also showed significantly higher sensitivity (65.7% vs. 32.1%) and a greater area under the ROC curve (0.94 vs. 0.62, *P* < 0.001) than UGI [21]. Likewise, a Japanese community-based study reported consistently higher sensitivity for EGD compared with UGI when assessed using both the detection and incidence methods. By the incidence method, sensitivity was 0.89 vs. 0.83 for prevalence screening and 0.95 vs. 0.86 for incidence screening, respectively [22]. Similarly, in China, where GC is common, there is no nationwide screening program, and UGI is rarely performed. Consequently, no region-based studies have directly compared the diagnostic accuracy of EGD and UGI. However, a large multicenter, regionally population-based cohort study conducted in six high-risk areas between 2005 and 2015 demonstrated the effectiveness of one-time EGD screening, showing a significant reduction in GC incidence compared with no screening (RR, 0.77; 95% CI, 0.74–0.81) [23]. Consistent with these findings from Japan, Korea, and China, a comprehensive meta-analysis synthesizing data from 10 population-based studies (six from Japan, two from Korea, and two from China) further reinforced these results, demonstrating a pooled sensitivity of 69.4% (95% CI, 61.0–77.8%) for EGD compared with 38.0% (95% CI, 27.0–49.1%) for UGI, yielding a relative detection rate of 2.8 (95% CI, 2.3–3.5), thereby strongly supporting the superior diagnostic yield of EGD for early GC detection [19].

A Japanese community-based case–control study found that individuals who underwent EGD screening within 36 months prior to diagnosis experienced a 30% reduction in GC mortality (OR, 0.70; 95% CI, 0.49–0.99), whereas UGI screening did not confer a statistically significant mortality benefit (OR, 0.87; 95% CI, 0.63–1.19) [18]. A meta-analysis further demonstrated that EGD screening in asymptomatic individuals was associated with an approximately 40% reduction in GC mortality compared with no screening [19].

One notable advantage of EGD over UGI is its ability to perform targeted biopsies during the procedure. This enables histopathological confirmation of suspicious lesions, as well as assessment of tumor histology and differentiation. Such information is essential for determining optimal treatment strategies and identifying premalignant or high-risk lesions at an early stage; such information cannot be obtained through UGI.

Taken together, evidence from high-prevalence countries such as Japan and Korea suggests that EGD is superior to UGI for asymptomatic individuals based on detection accuracy, GC-specific mortality reduction, safety profile, and cost-effectiveness. Although direct evidence from low- to moderate-prevalence countries remains limited, the available findings support the use of EGD rather than UGI for detecting early NCGC in asymptomatic individuals in these regions. Nevertheless, further studies evaluating mortality reduction and cost-effectiveness in low- to moderate-prevalence countries are warranted.

### Clinical question 3

Is risk stratification beneficial for detecting early NCGC in regions with low- to moderate-prevalence?

#### Statement

We suggest a risk stratification strategy to identify high-risk individuals for endoscopic detection of early NCGC in regions with low- to moderate-prevalence.

Certainty of evidence: Low.

Result of voting: 97.2% (Strongly agree, 69.4%; Agree, 27.8%).

#### Explanatory note

In low- to moderate-prevalence regions, no RCTs have directly evaluated whether risk-stratified screening improves the detection of early NCGC. In contrast, high-prevalence countries such as Japan and Korea have accumulated extensive epidemiological evidence supporting the concept of risk-based stratification for identifying individuals at elevated GC risk. These findings provide important contextual information when considering risk-stratified approaches in lower-prevalence settings. Noninvasive risk-stratification methods applicable before EGD in asymptomatic populations include *H. pylori* antibody testing, serum pepsinogen (PG) levels, their combined interpretation using the ABC method proposed by Miki et al., and other factors such as family history of GC, age, smoking, or dietary intake patterns [24, 25]. In addition, epidemiological characteristics, including country of birth or origin from a high-prevalence region, may provide important contextual information for individualized risk assessment, particularly in countries with increasingly diverse populations. Among these noninvasive indicators, serologic markers—particularly the combined assessment of *H. pylori* antibody and PG—are supported by the strongest body of evidence because they directly reflect the biological sequence from *H. pylori* infection to atrophic gastritis and subsequent gastric carcinogenesis. Other noninvasive factors, such as family history and lifestyle-related risks, provide supplementary predictive value but have lower individual discriminatory power.

A large Japanese cohort study evaluating the ABC classification—based on *H. pylori* antibody status and serum PG levels—demonstrated a clear stepwise increase in GC incidence across serologic groups [26]. These findings highlight the strong predictive value of combining serum PG levels and *H. pylori* status for risk stratification. In the Hisayama Study, serologic groups defined by *H. pylori* status and serum PG levels showed stepwise increases in GC incidence over more than 20 years of follow-up, with hazard ratios approaching 10 in the highest-risk group [27]. These long-term observations further demonstrate the robustness and reproducibility of serology-based risk stratification in a high-incidence setting. Korean population-based data also support the usefulness of serology-based risk stratification [28]. In these studies, serum PG II showed a strong and independent association with GC, particularly diffuse-type and early diffuse-type disease, even after adjustment for demographic and lifestyle factors. Furthermore, combining PG II levels with *H. pylori* status demonstrated a clear stepwise increase in GC risk.

Several limitations related to serologic markers should be considered. PG assays vary across countries and laboratories, and standardization remains inadequate. In addition, the performance of serology-based risk stratification depends heavily on the underlying prevalence of *H. pylori* infection. Because *H. pylori* seroprevalence varies widely across regions and has been declining globally, pretest probability may shift substantially, potentially affecting the predictive performance of serologic markers. A study from Sri Lanka—a country with low GC and *H. pylori* prevalence—showed that anti-*H. pylori* IgG and PG testing have good diagnostic performance for identifying *H. pylori* infection and gastric mucosal atrophy [29]. In this population, anti-*H. pylori* IgG demonstrated a sensitivity of 88.9% and a specificity of 91.6%, while the PG I/PG II ratio showed good ability to detect metaplasia (sensitivity, 83.3%; specificity, 95.4%) and moderate atrophy (sensitivity, 66.7%; specificity, 83.3%). However, this study did not evaluate GC outcomes; therefore, whether serology-based tools can be applied for GC risk stratification in low-prevalence settings remains uncertain and requires further validation.

When *H. pylori* infection is serologically suspected, several noninvasive factors—such as family history, dietary habits, and smoking—serve as useful complementary predictors of GC risk. Family history contributes additional risk information, with multiple case–control studies showing an approximately 2–3-fold increase in GC risk among individuals with an affected first-degree relative [30–32]. Dietary factors such as high salt intake or low dietary fiber intake have been consistently associated with GC risk across meta-analyses and prospectively collected cohort studies from various populations [33, 34]. Smoking is also an established risk factor, with evidence suggesting that its impact is particularly pronounced among individuals who are seropositive for *H. pylori* [35]. Although these familial and lifestyle factors can increase GC risk even in *H. pylori*-naive populations, such cases remain relatively uncommon; therefore, these variables primarily function as supplementary predictors in the context of suspected *H. pylori* infection.

Taken together, although absolute GC incidence differs substantially across regions, the RR gradients demonstrated by serologic stratification are consistent and robust in high-prevalence countries. Existing evidence supports *H. pylori* antibody and serum PG testing as the most evidence-based pre-endoscopic risk-stratification tools for asymptomatic individuals in low- to moderate-prevalence regions. Family history and lifestyle factors provide useful supplemental information but have lower individual predictive strength. At present, no established method for selecting candidates for endoscopic screening exists in these regions; however, serologic stratification represents the strongest available evidence-based approach.

### Clinical question 4

Can serological biomarkers assist in the identification of at-risk populations in low- to moderate-prevalence regions?

#### Statement

We suggest using serological biomarkers to identify individuals at high risk of NCGC in low- to moderate-prevalence regions.

Certainty of evidence: Low.

Result of voting: 94.3% (Strongly agree, 34.3%; Agree, 60.0%).

#### Explanatory note

Early identification of individuals at elevated risk of NCGC is essential for improving outcomes. Serological biomarkers are accessible tools for risk stratification, with *H. pylori* IgG antibodies and serum PG I/PG II levels being the most widely adopted [36]. *H. pylori* seropositivity—especially for virulence factors such as cytotoxin-associated gene A—is strongly associated with increased GC risk [37–40]. However, conventional enzyme-linked immunosorbent assays may underestimate prior infection due to declining antibody titers in individuals with advanced mucosal atrophy. Immunoblot assays offer improved sensitivity and have demonstrated significantly higher odds ratios for NCGC, underscoring the importance of assay selection in clinical risk assessment [41].

Serum pepsinogen levels, particularly PG I ≤ 70 ng/mL and PG I/PG II ≤ 3.0, are noninvasive indicators of atrophic gastritis, a precursor lesion for intestinal-type NCGC [42, 43]. Meta-analyses have reported moderate diagnostic accuracy for pepsinogen-based screening, with pooled sensitivity and specificity of approximately 69% and 73%, respectively, and an AUC of approximately 0.76 [44]. Combination models such as the ABC classification have demonstrated utility in population-based screening programs [26]. In this model, Group D (*H. pylori*-negative, PG-positive) reflects severe atrophy and carries the highest risk for NCGC, while Group B (*H. pylori*-positive, PG-negative) may be associated with diffuse-type cancers. Despite their utility, these approaches are limited by modest specificity and reduced sensitivity for certain histological subtypes, and they may be confounded by prior eradication therapy or proton pump inhibitor use.

Recent advances in molecular diagnostics have proposed circulating microRNAs (miRNAs) as highly promising biomarkers for GC screening. These small, stable RNA molecules are released into the bloodstream during tumorigenesis and reflect underlying gene regulatory changes. A multicenter study involving more than 5,000 subjects from Singapore and Korea developed and validated a 12-miRNA panel that achieved an AUC of 0.848, outperforming conventional blood-based markers, including *H. pylori* serology, PG ratios, carcinoembryonic antigen, and cancer antigen 19–9 [45].

Further supporting this approach, a recent meta-analysis encompassing 73 studies and more than 11,000 participants reported pooled sensitivity and specificity of 75% and 79%, respectively, with an AUC of 0.877 for circulating miRNAs. Subgroup analyses indicated that serum-based assays using U6 reference genes and SYBR Green-based quantitative PCR chemistry performed best, particularly in East Asian cohorts [46]. These findings suggest that miRNA-based assays may offer superior diagnostic performance and broader applicability across diverse populations. Taken together, the available evidence suggests that serological biomarkers may assist in the identification of individuals at increased risk of NCGC. While *H. pylori* serology and pepsinogen testing remain the most established and widely available approaches, emerging biomarkers such as circulating miRNAs may further improve risk stratification and support more targeted screening strategies in low- to moderate-prevalence regions, although additional validation is required before widespread implementation.

### Clinical question 5

Should oral preparations be used routinely pre-EGD for detecting early NCGC?

#### Statement

We recommend routine oral premedication with an antifoaming agent and a mucolytic 10–30 min prior to diagnostic EGD for early NCGC detection to improve gastric mucosal visibility.

Certainty of evidence: Moderate.

Result of voting: 100% (Strongly agree, 85.3%; Agree, 14.7%).

#### Explanatory note

Early NCGC and precancerous lesions often manifest as subtle, flat mucosal abnormalities that can be obscured by foam, mucus, or active peristalsis. Across multiple trials and a recent meta-analysis, oral premedication reliably improves mucosal cleanliness, most commonly assessed using the Total Mucosal Visibility Score (TMVS). Pooled data showed that mucolytics and antifoaming agents significantly improved visibility, with a mean TMVS reduction of 2.7 points. Simethicone alone also improved visibility, and some studies suggested that administration of 133 mg at least 20 min before EGD may provide optimal results [47].

Improved mucosal cleanliness is associated with reduced intraprocedural flushing and shorter examination times, potentially improving procedural efficiency. This may be particularly relevant in screening settings, given that preprocedure steps, including preparation and premedication, account for approximately 30% of total labor time in screening workflows [48]. Whether improved visibility consistently translates into increased neoplasm detection remains less clear. For antispasmodic agents, a large Korean propensity score-matched cohort study (*n* = 41,670) reported that cimetropium bromide was associated with an increased detection rate of gastric neoplasms (0.30% vs. 0.19%; adjusted OR, 1.42), particularly in the gastric body, which is relevant to NCGC [49]. In contrast, a Japanese retrospective cohort study (*n* = 31,484) evaluating other antispasmodics, including scopolamine, glucagon, and L-menthol, found no significant improvement in detection rates [50]. These findings suggest that the impact of antispasmodics on lesion detection may vary according to the specific agent used and examination conditions. Similarly, studies evaluating oral mucolytic and antifoaming preparations have shown heterogeneous results regarding lesion detection. A multicenter cluster-randomized study (*n* = 1,450) reported higher detection rates of precancerous lesions and early NCGC with pronase plus dimethicone compared with dimethicone alone (77.5% vs. 62.5%; OR, 3.84), together with shorter procedure times [51]. Conversely, another large multicenter RCT demonstrated that pronase plus sodium bicarbonate improved visibility and reduced flushing but did not significantly increase overall neoplasm detection compared with water alone [52]. Importantly, several studies suggest that improved mucosal cleanliness may be particularly beneficial when image-enhanced endoscopy (IEE) is used. Premedication with pronase has been reported to improve visibility in the proximal stomach and enhance the negative predictive value of narrow-band imaging (NBI) compared with white-light imaging alone [53, 54].

Taken together, the available evidence consistently supports the benefit of oral premedication for improving mucosal visibility and procedural efficiency during EGD. Although evidence of a direct increase in GC detection remains limited and heterogeneous across studies and regimens, improved mucosal visualization is an essential prerequisite for detecting subtle early NCGC and precancerous lesions. Therefore, given its favorable safety profile, low cost, and reproducible improvement in mucosal cleanliness, routine oral premedication with a mucolytic and an antifoaming agent 10–30 min before EGD is recommended to optimize gastric mucosal visualization and facilitate careful inspection for early NCGC.

### Clinical question 6

Does EGD under sedation improve the detection rate of early NCGC?

#### Statement

We suggest EGD under sedation to improve the detection of early NCGC by facilitating optimal conditions for high-quality endoscopic assessment.

Certainty of evidence: Moderate.

Result of voting: 100% (Strongly agree, 77.8%; Agree, 22.2%).

#### Explanatory note

The use of sedation during EGD is widely recommended by international clinical guidelines [55–58]. Sedation improves patient tolerance and reduces factors that may interfere with careful endoscopic examination, including discomfort, belching, nausea, and gastric peristalsis. In addition, sedation facilitates careful mucosal assessment and provides favorable conditions for high-quality endoscopic evaluation.

At present, however, no RCT has demonstrated that sedation directly improves lesion detection rates during EGD. Nevertheless, several observational studies have reported an association between sedated EGD and improved detection of upper gastrointestinal neoplastic lesions.

In a large multicenter retrospective study from China involving more than 400,000 diagnostic EGDs, the detection rate of gastric high-grade intraepithelial neoplasia and early GC was significantly higher in sedated than in unsedated examinations (0.16% vs. 0.12%, *P* = 0.020). In the same study, sedated EGD was also associated with longer examination times, more frequent use of image-enhanced and magnifying endoscopy, and a higher biopsy rate [59]. Similarly, a propensity score-matched retrospective study demonstrated that sedation was associated with a significantly higher detection rate of small upper gastrointestinal neoplastic lesions (≤ 10 mm) (2.80% vs. 2.02%, *P* = 0.001), including a higher detection rate of small malignant lesions (0.16% vs. 0.05%, *P* = 0.026) [60]. Although these findings suggest a beneficial association between sedation and neoplasm detection, they should be interpreted with caution. Both studies were observational and therefore susceptible to confounding. Importantly, factors known to influence lesion detection, such as observation time, use of advanced imaging techniques, endoscopist behavior, and biopsy practice, could not be fully controlled. Consequently, the observed association between sedation and lesion detection may be influenced, at least in part, by improvements in examination quality rather than by a direct effect of sedation itself. Sedation may enhance patient tolerance and cooperation, facilitate adequate gastric distension, and enable more stable and meticulous mucosal inspection, thereby facilitating high-quality endoscopic assessment.

Furthermore, recent studies have demonstrated an association between sedation status and the risk of missed GC. Nonsedated EGD has been identified as a risk factor for missed GC, whereas intravenous sedation has been associated with a reduced risk of missed GC [59, 61]. Although these observations do not establish a direct causal effect of sedation on lesion detection, they further support the notion that sedation may enhance diagnostic performance by facilitating high-quality endoscopic examination.

Taken together, the available evidence does not support a direct effect of sedation on lesion detection. Rather, sedation appears to facilitate high-quality endoscopic assessment, which may indirectly contribute to improved detection of precancerous lesions and early GC. However, because the current evidence is derived primarily from observational studies, prospective studies are warranted to clarify the causal impact of sedation on lesion detection during EGD.

### Clinical question 7

What is the appropriate inspection time for EGD to improve the detection of early NCGC?

#### Statement

We suggest a total examination time of at least 5 min for systematic and comprehensive gastric mucosal inspection to improve the detection of early NCGC and ensure the quality of EGD.

Certainty of evidence: Low.

Result of voting: 100% (Strongly agree, 79.4%; Agree, 20.6%).

#### Explanatory note

Prolonged inspection times during EGD are generally associated with improved detection rates of clinically significant gastric lesions. However, practical constraints in clinical workflows limit procedure duration, as extended examination times increase sedation requirements, reduce patient tolerability, and impose substantial resource burdens on endoscopy units. Consequently, several studies have sought to determine the optimal duration of EGD that balances detection efficacy with procedural feasibility.

A retrospective cohort study from Japan evaluated the impact of examination time on the detection of neoplastic lesions during EGD performed without biopsy. Endoscopists with moderate procedure durations (5–7 min) detected approximately twice as many neoplastic lesions as faster operators (< 5 min) (OR, 1.90; 95% CI, 1.06–3.40). Notably, operators with examination times exceeding 7 min did not demonstrate further improvement in detection rates beyond the moderate-duration group [62].

In a retrospective single-center study from Singapore, endoscopists with mean EGD durations exceeding 7 min achieved significantly higher detection rates of high-risk lesions (OR, 2.50; 95% CI, 1.52–4.12) and neoplastic lesions (OR, 3.42; 95% CI, 1.25–10.38) compared with those with shorter procedure times [63]. It is important to note, however, that in this study, the total EGD duration was measured from the time of endoscope insertion to withdrawal, including biopsy, suggesting that the actual mucosal inspection time was less than 7 min.

Similarly, Park et al. analyzed data from a 7-year health screening program, focusing specifically on withdrawal time as a proxy for inspection duration. Their findings corroborated that slower endoscopists had higher detection rates of gastric adenomas and carcinomas [64]. More recently, a single-center retrospective study from China reported that an observation time of ≥5 min was associated with a higher detection rate of focal gastric lesions [65]. Another Korean study found that patients with multiple neoplasms diagnosed during a single EGD session had significantly longer examination times than those in whom additional lesions were identified during follow-up EGDs (6.5 ± 2.4 min vs. 3.8 ± 1.8 min; *P* < 0.001) [66].

These findings collectively underscore the critical role of adequate examination time in optimizing early detection of gastric neoplasia. As such, examination duration should be considered a core quality metric in EGD performance assessment. A systematic and comprehensive evaluation of the upper gastrointestinal tract—incorporating acquisition of a standardized minimum number of images—should ideally allocate at least 5 min for mucosal inspection to ensure high-quality diagnostic performance. Further studies are therefore needed to determine the optimal examination time specifically for the detection of early GC.

### Clinical question 8

Should systematic photodocumentation with landmarks in EGD be standardized to improve the detection rate of early NCGC?

#### Statement

We suggest standardizing systematic photodocumentation with landmarks in EGD to improve the detection of early NCGC.

Certainty of evidence: Low.

Result of voting: 100% (Strongly agree, 74.3%; Agree, 25.7%).

#### Explanatory note

Early detection of NCGC significantly improves patient prognosis, but subtle lesions are often difficult to identify without systematic examination. To date, no RCTs have investigated the effectiveness of systematic photodocumentation during screening EGD; however, several retrospective studies from Japan and South Korea have emphasized the importance of structured inspection with systematic photodocumentation.

Japanese researchers proposed the Systematic Screening Protocol for the Stomach (SSS protocol). The SSS protocol defines 22 key images covering all gastric regions in both antegrade and retroflex views to comprehensively map the stomach and minimize blind spots [67]. In a retrospective observational study from South Korea involving approximately 6,000 EGDs, endoscopists who obtained a larger number of images according to the SSS protocol demonstrated higher detection rates of clinically significant gastric lesions, and the high-photodocumentation group showed significantly higher lesion detection rates than the low-photodocumentation group (adjusted OR, 2.07; 95% CI, 1.41–3.05) [68]. Another Japanese retrospective study demonstrated that the number of images is a key quality indicator in screening EGD [69]. Capturing < 50 total images significantly reduced overall cancer detection, while obtaining < 35 gastric images markedly lowered GC detection. A trend toward reduced detection was also observed when < 11 esophageal images were obtained. The findings support using ≥ 50 total images, including ≥ 35 gastric images, to optimize upper gastrointestinal cancer detection.

These data support the concept that increasing the number of images correlates with improved detection of clinically relevant lesions, including early GC. The European Society of Gastrointestinal Endoscopy (ESGE) guidelines recommend a minimum of 8–10 key landmark images, reinforcing the importance of completeness in the examination [69, 70, 70, 71] In these guidelines, systematic photodocumentation ensures that the entire upper gastrointestinal tract—esophagus, stomach, and duodenum—is carefully inspected and documented, reducing the likelihood of missed lesions.

Even in regions with a low- to moderate-prevalence of GC, high-quality EGD screening is expected to contribute to improved GC detection rates; therefore, systematic photodocumentation techniques should also be standardized in these regions. Implementation challenges in low-resource environments necessitate a pragmatic approach focusing on thorough mucosal visualization, even if not all recommended images can be captured and stored [54].

### Clinical question 9

Does IEE improve the detection of early NCGC?

#### Statement

We suggest the use of IEE, in addition to white-light endoscopy (WLE), for detecting early NCGC in high-risk individuals, although effectiveness varies across imaging modalities.

Certainty of evidence: Moderate.

Result of voting: 100% (Strongly agree, 75.0%; Agree, 25.0%).

#### Explanatory note

The diagnosis of early GC by EGD can be divided into two steps: endoscopic lesion detection, followed by lesion characterization to establish the diagnosis. Historically, lesion detection was achieved using conventional WLE. Over the years, various image-enhancement technologies have been developed, such as NBI (Olympus Medical), Texture and Color Enhancement (TXI, Olympus Medical), Linked Color Imaging (LCI, Fujifilm Corporation), Blue Laser Imaging (BLI, Fujifilm Corporation), and Optical Enhancement (OE, Pentax Corporation). These technologies utilize specialized light-source modifications, magnification, or image-processing techniques to enhance structural changes on the mucosal surface, with the aim of improving the diagnosis of early gastrointestinal pathologies. To date, no RCTs have evaluated the effectiveness of IEE for GC detection and diagnosis in low- to moderate-prevalence regions.

NBI and BLI both use specific narrow wavelengths of light to highlight mucosal surface changes, which can result in a slightly darker overall image. An early RCT of 579 average-risk patients from across Asia comparing second-generation NBI with WLE showed a higher focal gastric lesion detection rate; however, this finding was attributed to increased detection of intestinal metaplasia rather than GC [72]. A larger multicenter Japanese randomized study involving 4,513 high-risk patients failed to demonstrate the superiority of second-generation NBI for improving GC detection [73]. The study showed low GC event rates of 1.9% and 2.3%, although the positive predictive value for suspicious lesions was higher with NBI than with WLE. In contrast, a two-center Japanese randomized trial of 596 high-risk patients showed that BLI-bright significantly increased the GC detection rate compared with WLE [74]. Of note, the GC event rate in this study was higher than the previously reported rate, at 7.9% (47/596 patients). Kadota et al. recently reported in a three-arm randomized phase II trial of 901 patients that the detection rate of GC was highest with third-generation NBI (7.3%) compared with TXI (5.0%) and WLE (5.6%) [75]. A meta-analysis of three studies utilizing NBI technology concluded that NBI did not increase the gastric neoplasm detection rate; however, the results should be interpreted with caution because of the heavy weighting toward second-generation NBI studies and the small sample size evaluating the latest third-generation technology [76].

In an RCT of 2,383 high-risk patients from China, Gao et al. reported that the addition of LCI to WLE significantly increased the GC detection rate from 4.31% to 8.01% [77]. Similarly, Ono et al. reported superior performance of LCI over WLE in detecting early upper gastrointestinal neoplasms among high-risk patients in a multicenter Japanese randomized study [78]. A recent multicenter RCT showed that LCI alone remained superior to WLE for detecting early GC, particularly differentiated-type adenocarcinoma [79]. The benefit of LCI over WLE in detecting early GC was also demonstrated in a recent meta-analysis comprising more than 7,000 patients [80]. In another recent RCT, OE mode 2 was also found to improve the gastric neoplasia detection rate from 1.9% to 5.1% compared with WLE [81].

Interestingly, a randomized study directly compared LCI with second-generation NBI for surveillance of high-risk individuals and found no difference in detection rates between the two technologies. However, the percentage of WLE-detected gastric epithelial neoplasms was significantly higher in the NBI group than in the LCI group (3.0% vs. 0.27%) [82]. These findings suggest possible baseline differences in WLE performance, even among endoscopic systems from different manufacturers.

In conclusion, in a selected high-risk population, the use of IEE may improve GC detection rates.

### Clinical question 10

Can endoscopic imaging accurately assess gastric atrophy or intestinal metaplasia to help identify high-risk patients for early NCGC?

#### Statement

We suggest confirming endoscopic suspicion of gastric atrophy and intestinal metaplasia histologically by biopsy in regions with a low- to moderate-prevalence.

Certainty of evidence: Low.

Result of voting: 97.1% (Strongly agree, 44.1%; Agree, 52.9%).

#### Explanatory note

Gastric atrophy and intestinal metaplasia are well-recognized precancerous conditions that typically arise as consequences of *H. pylori* infection. The gold standard for assessing these lesions is histological evaluation of biopsy specimens, most commonly using the Updated Sydney System (USS), which provides a standardized grading system for gastritis, atrophy, and intestinal metaplasia in both the antrum and corpus. Building on this framework, the Operative Link on Gastritis Assessment (OLGA) and the Operative Link on Gastric Intestinal Metaplasia Assessment (OLGIM) are available histological staging systems [83–85]. More recently, the Operative Link on Gastric Intestinal Metaplasia and Glandular Atrophy Assessment (OLGIMA) has also been proposed [86]. The severity of atrophy or intestinal metaplasia, as graded by these systems, has been shown to correlate with GC risk. Meta-analyses have demonstrated that high-stage (stage III/IV) disease according to OLGA or OLGIM is significantly associated with increased GC risk, supporting the utility of these staging systems in identifying individuals who may require closer endoscopic follow-up [87]. Several reports from both high- and low- to moderate-prevalence regions have demonstrated the diagnostic yield of endoscopy for gastric atrophy and intestinal metaplasia.

The diagnostic performance of endoscopic assessment for gastric atrophy and intestinal metaplasia has been evaluated in several studies using histology from biopsy specimens as the reference standard. For gastric atrophy, the Kimura–Takemoto classification is commonly used for endoscopic grading. In a study of Japanese patients, the agreement between endoscopic atrophy grading using the Kimura–Takemoto classification and histological atrophy scores according to the USS showed a weighted κ value of 0.51 (95% CI, 0.44–0.59) [88]. Another study compared the extent of atrophy graded by WLE using a modified Kimura–Takemoto classification with histological atrophy scores from five biopsy sites according to the USS, reporting a weighted κ value of 0.76 (*P* < 0.001) [89]. In Vietnamese patients with dyspepsia, endoscopic gastric atrophy graded by the Kimura–Takemoto classification showed a moderate positive correlation with histological OLGA stage (*r* = 0.6). Although moderate-to-severe atrophy identified all patients with a high OLGA stage (sensitivity and negative predictive value, both 100%), its limited specificity (57.7%) indicates a risk of overestimating high-risk cases [90]. Taken together, findings from these studies suggest that endoscopic and histological assessments of gastric atrophy show moderate-to-substantial agreement, although endoscopy alone may overestimate risk in specific populations.

In contrast, the diagnostic accuracy of conventional WLE for intestinal metaplasia is insufficient. In a Korean study involving 1,333 patients, the sensitivity based on histological assessment was only 24.0% for the antrum and 24.2% for the corpus, while the specificity was 91.9% and 88.0%, respectively [91]. Even in a prospective study using high-definition WLE, the overall sensitivity improved to 74.6%, and all significant advanced intestinal metaplasia and dysplastic lesions were detected; however, mild intestinal metaplasia was often not endoscopically visible, and its detectability remained limited [92]. Thus, while endoscopic findings of intestinal metaplasia show relatively high specificity and can reliably detect clinically significant lesions, their low sensitivity means that mild lesions are often missed, and reliance on endoscopy alone may underestimate risk.

Recent studies have demonstrated that IEE improves detection of precancerous gastric conditions compared with WLE. For intestinal metaplasia, the Endoscopic Grading of Gastric Intestinal Metaplasia (EGGIM) score under NBI correlates strongly with the histological OLGIM stage, with an EGGIM score ≥ 5 predicting high-stage OLGIM with a sensitivity of 89–94% and specificity of 91–95% [93]. Similarly, the EGGIM score using LCI has also been validated, with a cutoff value of 4 showing high diagnostic performance for OLGIM stage III/IV (sensitivity, 94.1%; specificity, 86.4%) [94]. Collectively, these findings suggest that IEE may provide high sensitivity and specificity for the endoscopic diagnosis of intestinal metaplasia. Based on the current evidence, endoscopic findings for gastric atrophy show high sensitivity but low specificity, which may lead to overestimation of risk. In contrast, for intestinal metaplasia, endoscopic findings show relatively high specificity but low sensitivity, raising the possibility of risk underestimation. Although IEE may compensate for some of the limitations of WLE in detecting intestinal metaplasia, its diagnostic performance is insufficient to justify reliance on endoscopy alone. Therefore, when atrophy is suspected endoscopically, risk stratification should be performed using histological staging according to the USS or OLGA. The Management of Epithelial Precancerous Conditions and Early Neoplasia of the Stomach (MAPS III) guideline suggests that biopsy sampling may be simplified when advanced mucosal atrophic changes, such as severe atrophy of C-3 or higher and/or an EGGIM score ≥ 5, are identified endoscopically [95]. However, in regions with low to intermediate GC risk, the positive predictive value of these endoscopic findings may be limited [93]. Therefore, this consensus placed greater emphasis on negative predictive value and concluded that histological confirmation is desirable when endoscopic findings raise suspicion of gastric atrophy or intestinal metaplasia. Endoscopic findings alone should not be used to evaluate intestinal metaplasia.

### Clinical question 11

Should artificial intelligence (AI) endoscopy be used for improving the detection rate of early NCGC in low- to moderate-prevalence regions?

#### Statement

We could not reach consensus on whether to recommend or not recommend the routine use of AI-assisted endoscopy to improve the detection of early NCGC in low- to moderate-prevalence regions.

Certainty of evidence: Low.

Result of voting: 65.7% (Strongly agree, 37.1%; Agree, 28.6%).

#### Explanatory note

Improving the detection of early GC is a global priority in EGD, and several technologies, including AI, have been widely adopted. Currently, several AI technologies are available depending on their intended purpose; for example, computer-aided detection for detection assistance, computer-aided diagnosis for lesion characterization, and AI-based systems for quality assurance. Although many studies have shown that AI can detect GC as well as or better than experienced endoscopists, most have focused on diagnostic performance, and multiple confounding factors may limit inferences regarding real-world benefits, especially in low- to moderate-prevalence regions.

Early GC lesions are often small, flat, and subtle, making them difficult to detect endoscopically. Interoperator variation in the completeness of mucosal inspection further affects detection rates. Several reports from China have demonstrated the effectiveness of AI-driven quality assurance systems. Initially, Wu et al. conducted a single-center, prospective, parallel-group trial and found that AI reduced the blind-spot rate (5.86% vs. 22.46%, *P* < 0.001) [96]. AI assistance was also associated with longer examination times (5.03 vs. 4.24 min, *P* < 0.001) and more complete photodocumentation (90.64% vs. 79.14%, *P* < 0.001). A multicenter study across five Chinese hospitals by the same group reported fewer blind spots (5.38 vs. 9.82, *P* < 0.001) and longer inspection times (5.40 vs. 4.38 min, *P* < 0.001) with AI, although these studies emphasized procedural completeness but did not report correlations with gastric neoplasm detection [97]. Li et al. reported a positive correlation between an AI-derived completeness score and detection rates for upper gastrointestinal cancer, including early cancers (*r* = 0.775, *P* < 0.003; *r* = 0.756, *P* < 0.004, respectively) [98]. Although this analysis included all upper gastrointestinal cancers, the stomach accounted for the majority, and approximately half of the early cancers were GC, suggesting that AI-assisted quality assessment may contribute to improved GC detection. Conversely, improved inspection alone may not translate into higher neoplasm detection in low- to moderate-prevalence regions. In a prospective sequential cohort study conducted in Hong Kong by Chan et al., eight trainees using real-time AI achieved a higher completeness rate (92.6% vs. 71.2%, *P* < 0.001) with similar inspection times (765.5 s vs. 740.4 s, *P* = 0.374), but no significant difference in pathology-confirmed cases was observed (3.43% vs. 2.16%, *P* = 0.399) [99]. However, the lack of data from low- to moderate-prevalence regions limits the generalizability of these findings.

In a single-center randomized tandem trial focusing on gastric neoplasm detection technology, Wu et al. reported a lower gastric neoplasm miss rate with real-time AI (6.1% vs. 27.3%, *P* = 0.015). The incremental detections were primarily lesions < 10 mm and low-grade dysplasia rather than carcinoma or high-grade dysplasia [100]. The overall gastric neoplasm detection rate in that study (3.5–4.9%) was higher than typical, and observer bias from the same endoscopist may have influenced the results. Another large-scale prospective, single-blinded, multicenter RCT by Zhou et al. demonstrated higher upper gastrointestinal tumor detection (6.1% vs. 2.3%, *P* = 0.0001), including early GC, and reduced blind-spot rates (2.3 vs. 6.2, *P* < 0.001). This study suggested that combining AI detection with systematic quality measurement yields benefits across centers, endoscopist experience levels, and all dysplastic lesions [101]. Unfortunately, several studies have failed to demonstrate a clear clinical benefit. An open-label, single-center RCT from Korea by Gong et al. found a nonstatistically significant increase in lesion detection when AI was added to examinations performed by experienced endoscopists (2.0% vs. 1.3%, *P* = 0.210) [102]. Retrospective data from Huang et al. across two institutions showed institution-specific effects: hospitals with low baseline biopsy rates reported higher gastric neoplasm detection (OR, 1.84; *P* < 0.001) but no significant increase in early GC detection; hospitals with high biopsy rates showed increased early GC detection without a change in overall neoplasm detection [103]. A retrospective Japanese study by Adachi et al. did not demonstrate a difference in overall upper gastrointestinal tumor rates (1.02% vs. 0.63%, *P* = 0.062) but reported a higher gastric tumor rate (0.68% vs. 0.32%, *P* = 0.024), potentially reflecting the effects of certified endoscopists, higher biopsy rates, and longer examination times [104]. Similarly, a prospective randomized pretest–posttest trial by Xu et al. found increased detection of precancerous gastric lesions (14.23% vs. 9.15%, *P* = 0.013) but not early GC [105].

Thus, most positive data have been derived from high-prevalence settings or specific circumstances, including operator experience, biopsy and sedation practices, and outcome definitions. In summary, AI can improve measurable quality metrics for EGD; however, evidence that it reliably increases the detection of early NCGC in low- to moderate-prevalence regions remains inconclusive. Accordingly, although consensus was not achieved for this recommendation, the panel considered AI to be a rapidly evolving field with substantial potential to improve GC screening. Further prospective studies and external validation in low- to moderate-prevalence regions are warranted before a definitive recommendation can be established.

## Discussion

This international consensus provides recommendations for GC screening and early detection in low- to moderate-prevalence regions, where optimal screening strategies remain uncertain. Overall, the consensus does not support routine population-based endoscopic screening for asymptomatic individuals because of insufficient direct evidence. However, when GC screening is performed, EGD is preferred over UGI, and risk-stratified approaches may help identify individuals who are more likely to benefit from endoscopic screening. The consensus also emphasizes the importance of high-quality EGD, including adequate mucosal preparation, appropriate examination conditions, sufficient inspection time, systematic photodocumentation, and the appropriate use of advanced imaging and artificial intelligence-assisted technologies. Detailed discussion of the evidence and limitations for each recommendation is provided in the corresponding CQ sections.

A major strength of this consensus is the participation of international experts from diverse geographic regions and the use of a structured consensus process to address a broad range of clinically relevant issues. The 11 CQs cover key aspects of GC screening and early detection, ranging from screening strategies and risk stratification to endoscopy quality and advanced technologies. This broad scope provides a practical framework for considering GC screening and early detection in low- to moderate-prevalence regions.

Several important limitations should be acknowledged. Most importantly, direct evidence from low- to moderate-prevalence regions remains limited, and many recommendations are based largely on evidence from high-prevalence regions, particularly East Asia, together with expert judgment. Differences in GC incidence, *H. pylori* prevalence, healthcare systems, endoscopic resources, and screening practices may limit the direct applicability of these findings to low- to moderate-prevalence settings. In addition, the quantity and quality of available evidence varied across CQs, and the certainty of evidence for several recommendations was limited. Further studies in low- to moderate-prevalence regions are needed to validate these recommendations and to develop screening strategies appropriate to local populations and healthcare systems.

## Supplementary Information

Below is the link to the electronic supplementary material.

Supplementary file1

## Data Availability

Data are available from the corresponding author upon request.

## References

[1] Bray F, Laversanne M, Sung H, Ferlay J, Siegel RL, Soerjomataram I, et al. Global cancer statistics 2022: GLOBOCAN estimates of incidence and mortality worldwide for 36 cancers in 185 countries. CA Cancer J Clin. 2022;74:229–63. 10.3322/caac.21834 38572751

[2] Thrift AP, Wenker TN, El-Serag HB. Global burden of gastric cancer: epidemiological trends, risk factors, screening and prevention. Nat Rev Clin Oncol. 2023;20:338–49. 10.1038/s41571-023-00747-0 36959359

[3] Rawla P, Barsouk A. Epidemiology of gastric cancer: global trends, risk factors and prevention. Gastroenterol Rev. 2019;14:26–38. 10.5114/pg.2018.80001 PMC6444111 30944675

[4] Wang C, Nishiyama T, Kikuchi S, Inoue M, Sawada N, Tsugane S, et al. Changing trends in the prevalence of H. pylori infection in Japan (1908-2003): a systematic review and meta-regression analysis of 170,752 individuals. Sci Rep. 1908;7:15491. 10.1038/s41598-017-15490-7 PMC5686167 29138514

[5] Ishibashi F, Ichita C, Takasu A, Li R, Hagiwara Y, Matsuyama Y, et al. The increasing trend of the generational *Helicobacter pylori*-naïve prevalence among Japanese individuals born between 1925 and 2015: a systematic review and meta-regression analysis. Helicobacter. 1925;30:e70052. 10.1111/hel.70052 40500921

[6] Matsuzaka M, Di Carlo V, Matz M, Sugiyama H, Oki I, Shibata A, et al. Trends in net survival for patients with stomach cancer in Japan, 2000-14 (CONCORD-3). Jpn J Clin Oncol. 2000;56:i25–33. 10.1093/jjco/hyaf130 41859881

[7] Imamura Y, Watanabe M, Oki E, Morita M, Baba H. Esophagogastric junction adenocarcinoma shares characteristics with gastric adenocarcinoma: literature review and retrospective multicenter cohort study. Ann Gastroenterol Surg. 2021;5:46–59. 10.1002/ags3.12406 PMC7832959 33532680

[8] Guyatt GH, Oxman AD, Vist GE, Kunz R, Falck-Ytter Y, Alonso-Coello P, et al. GRADE: an emerging consensus on rating quality of evidence and strength of recommendations. BMJ. 2008;336:924–6. 10.1136/bmj.39489.470347.AD PMC2335261 18436948

[9] Fock KM, Ang TL. Epidemiology of *Helicobacter pylori* infection and gastric cancer in Asia. J Gastroenterol Hepatol. 2010;25:479–86. 10.1111/j.1440-1746.2009.06188.x 20370726

[10] Ang TL, Fock KM. Clinical epidemiology of gastric cancer. Singapore Med J. 2014;55:621–8. 10.11622/smedj.2014174 PMC4291998 25630323

[11] Kong SH, Park DJ, Lee HJ, Jung HC, Lee KU, Choe KJ, et al. Clinicopathologic features of asymptomatic gastric adenocarcinoma patients in Korea. Jpn J Clin Oncol. 2004;34:1–7. 10.1093/jjco/hyh009 15020656

[12] Matsukuma A, Furusawa M, Tomoda H, Seo Y. A clinicopathological study of asymptomatic gastric cancer. Br J Cancer. 1996;74:1647–50. 10.1038/bjc.1996.603 PMC2074857 8932349

[13] Berrill JW, Turner JK, Hurley JJ, Swift G, Dolwani S, Green JT. Upper gastrointestinal cancer in its early stages is predominantly asymptomatic. Frontline Gastroenterol. 2012;3:47–51. 10.1136/flgastro-2011-100026 PMC5517248 28839631

[14] Kim BJ, Heo C, Kim BK, Kim JY, Kim JG. Effectiveness of gastric cancer screening programs in South Korea: organized vs opportunistic models. World J Gastroenterol. 2013;19:736–41. 10.3748/wjg.v19.i5.736 PMC3574600 23430471

[15] Choi KS, Jun JK, Suh M, Park B, Noh DK, Song SH, et al. Effect of endoscopy screening on stage at gastric cancer diagnosis: results of the National Cancer Screening Programme in Korea. Br J Cancer. 2015;112:608–12. 10.1038/bjc.2014.608 PMC4453643 25490528

[16] Narii N, Sobue T, Zha L, Kitamura T, Iwasaki M, Inoue M, et al. Effectiveness of endoscopic screening for gastric cancer: the Japan Public Health Center-based Prospective Study. Cancer Sci. 2022;113:3922–31. 10.1111/cas.15545 PMC9633299 36002149

[17] Jun JK, Choi KS, Lee HY, Suh M, Park B, Song SH, et al. Effectiveness of the Korean National Cancer Screening Program in reducing gastric cancer mortality. Gastroenterology. 2017;152:1319-28.e7. 10.1053/j.gastro.2017.01.029 28147224

[18] Hamashima C, Ogoshi K, Okamoto M, Shabana M, Kishimoto T, Fukao A. A community-based, case-control study evaluating mortality reduction from gastric cancer by endoscopic screening in Japan. PLoS ONE. 2013;8:e79088. 10.1371/journal.pone.0079088 PMC3827316 24236091

[19] Zhang X, Li M, Chen S, Hu J, Guo Q, Liu R, et al. Endoscopic screening in Asian countries is associated with reduced gastric cancer mortality: a meta-analysis and systematic review. Gastroenterology. 2018;155:347-54.e9. 10.1053/j.gastro.2018.04.026 29723507

[20] Yim JY, Kim N, Choi SH, Kim YS, Cho KR, Kim SS, et al. Seroprevalence of *Helicobacter pylori* in South Korea. Helicobacter. 2007;12:333–40. 10.1111/j.1523-5378.2007.00504.x 17669107

[21] Choi KS, Jun JK, Park EC, Park S, Jung KW, Han MA, et al. Performance of different gastric cancer screening methods in Korea: a population-based study. PLoS ONE. 2012;7:e50041. 10.1371/journal.pone.0050041 PMC3510189 23209638

[22] Hamashima C, Okamoto M, Shabana M, Osaki Y, Kishimoto T. Sensitivity of endoscopic screening for gastric cancer by the incidence method. Int J Cancer. 2013;133:653–9. 10.1002/ijc.28065 23364866

[23] Chen R, Liu Y, Song G, Li B, Zhao D, Hua Z, et al. Effectiveness of one-time endoscopic screening programme in prevention of upper gastrointestinal cancer in China: a multicentre population-based cohort study. Gut. 2021;70:251–60. 10.1136/gutjnl-2019-320200 PMC7815635 32241902

[24] Miki K. Gastric cancer screening by combined assay for serum anti-*Helicobacter pylori* IgG antibody and serum pepsinogen levels - “ABC method.” Proc Jpn Acad Ser B Phys Biol Sci. 2011;87:405–14. 10.2183/pjab.87.405 PMC3171284 21785258

[25] Miki K. Gastric cancer screening using the serum pepsinogen test method. Gastric Cancer. 2006;9:245–53. 10.1007/s10120-006-0397-0 17235625

[26] Watabe H, Mitsushima T, Yamaji Y, Okamoto M, Wada R, Kokubo T, et al. Predicting the development of gastric cancer from combining *Helicobacter pylori* antibodies and serum pepsinogen status: a prospective endoscopic cohort study. Gut. 2005;54:764–8. 10.1136/gut.2004.055400 PMC1774550 15888780

[27] Ikeda F, Shikata K, Hata J, Fukuhara M, Hirakawa Y, Ohara T, et al. Combination of helicobacter pylori antibody and serum pepsinogen as a good predictive tool of gastric cancer incidence: 20-year prospective data from the hisayama study. J Epidemiol. 2016;26:629–36. 10.2188/jea.JE20150258 PMC5121431 27265836

[28] Lim SH, Kim N, Choi Y, Choi JM, Han YM, Kwak MS, et al. Assessing serum pepsinogen and *Helicobacter pylori* tests for detecting diffuse-type gastric cancer: insights from a large-scale and propensity-score-matched study in Republic of Korea. Cancers (Basel). 2025. 10.3390/cancers17060955. PMC11940262 40149291

[29] Doohan D, Fauzia KA, Rathnayake J, Lamawansa MD, Waskito LA, Tuan VP, et al. Pepsinogen and serum IgG detection is a valuable diagnostic method for *Helicobacter pylori* infection in a low-prevalence country: a report from Sri Lanka. Diagnostics (Basel). 2021. 10.3390/diagnostics11081364. PMC8391933 34441303

[30] Dhillon PK, Farrow DC, Vaughan TL, Chow WH, Risch HA, Gammon MD, et al. Family history of cancer and risk of esophageal and gastric cancers in the United States. Int J Cancer. 2001;93:148–52. 10.1002/ijc.1294 11391635

[31] La Vecchia C, Negri E, Franceschi S, Gentile A. Family history and the risk of stomach and colorectal cancer. Cancer. 1992;70:50–5. 10.1002/1097-0142(19920701)70:1<50::aid-cncr2820700109>3.0.co;2-i 1606546

[32] Minami Y, Tateno H. Associations between cigarette smoking and the risk of four leading cancers in Miyagi Prefecture, Japan: a multi-site case-control study. Cancer Sci. 2003;94:540–7. 10.1111/j.1349-7006.2003.tb01480.x PMC11160141 14529588

[33] Wu B, Yang D, Yang S, Zhang G. Dietary salt intake and gastric cancer risk: a systematic review and meta-analysis. Front Nutr. 2021;8:801228. 10.3389/fnut.2021.801228 PMC8692376 34957192

[34] Takasu A, Gotoda T, Suzuki S, Kusano C, Goto C, Ishikawa H, et al. Daily diet and nutrition risk factors for gastric cancer incidence in a Japanese population. Gut Liver. 2024;18:602–10. 10.5009/gnl230354 PMC11249943 38388181

[35] Butt J, Varga MG, Wang T, Tsugane S, Shimazu T, Zheng W, et al. Smoking, *Helicobacter pylori* serology, and gastric cancer risk in prospective studies from China, Japan, and Korea. Cancer Prev Res (Phila). 2019;12:667–74. 10.1158/1940-6207.CAPR-19-0238 PMC6854526 31350279

[36] Aalami AH, Aalami F, Sahebkar A. Gastric cancer and circulating microRNAs: an updated systematic review and diagnostic meta-analysis. Curr Med Chem. 2023;30:3798–814. 10.2174/0929867330666221121155905 36411580

[37] Bang CS, Lee JJ, Baik GH. Prediction of chronic atrophic gastritis and gastric neoplasms by serum pepsinogen assay: a systematic review and meta-analysis of diagnostic test accuracy. J Clin Med. 2019. 10.3390/jcm8050657. PMC6572271 31083485

[38] Huang YK, Yu JC, Kang WM, Ma ZQ, Ye X, Tian SB, et al. Significance of serum pepsinogens as a biomarker for gastric cancer and atrophic gastritis screening: a systematic review and meta-analysis. PLoS ONE. 2015;10:e0142080. 10.1371/journal.pone.0142080 PMC4640555 26556485

[39] In H, Sarkar S, Ward J, Friedmann P, Parides M, Yang J, et al. Serum pepsinogen as a biomarker for gastric cancer in the United States: a nested case-control study using the PLCO cancer screening trial data. Cancer Epidemiol Biomarkers Prev. 2022;31:1426–32. 10.1158/1055-9965.EPI-21-1328 PMC9268394 35534235

[40] Inoue M, Sawada N, Goto A, Shimazu T, Yamaji T, Iwasaki M, et al. High-negative anti-*Helicobacter pylori* IgG antibody titers and long-term risk of gastric cancer: results from a large-scale population-based cohort study in Japan. Cancer Epidemiol Biomarkers Prev. 2020;29:420–6. 10.1158/1055-9965.EPI-19-0993 31826914

[41] Kishikawa H, Kimura K, Takarabe S, Kaida S, Nishida J. *Helicobacter pylori* antibody titer and gastric cancer screening. Dis Markers. 2015. 10.1155/2015/156719. PMC4606161 26494936

[42] Murphy JD, Epplein M, Lin FC, Troester MA, Nichols HB, Butt J, et al. Discrimination between precancerous gastric lesions and gastritis using a gastric cancer risk stratification model. Asian Pac J Cancer Prev. 2023;24:935–43. 10.31557/APJCP.2023.24.3.935 PMC10334080 36974548

[43] So JBY, Kapoor R, Zhu F, Koh C, Zhou L, Zou R, et al. Development and validation of a serum microRNA biomarker panel for detecting gastric cancer in a high-risk population. Gut. 2021;70:829–37. 10.1136/gutjnl-2020-322065 PMC8040159 33028667

[44] Tatemichi M, Sasazuki S, Inoue M, Tsugane S. Clinical significance of IgG antibody titer against *Helicobacter pylori*. Helicobacter. 2009;14:231–6. 10.1111/j.1523-5378.2009.00681.x 19702853

[45] Fan XH, Zhang Y, Wang P, Song QQ, Wang M, Mejias-Luque R, et al. A noninvasive multianalytical approach establishment for risk assessment and gastric cancer screening. Int J Cancer. 2024;154:1111–23. 10.1002/ijc.34739 37842828

[46] González CA, Megraud F, Buissonniere A, Lujan Barroso L, Agudo A, Duell EJ, et al. *Helicobacter pylori* infection assessed by ELISA and by immunoblot and noncardia gastric cancer risk in a prospective study: the Eurgast-EPIC project. Ann Oncol. 2012;23:1320–4. 10.1093/annonc/mdr384 21917738

[47] Burke E, Harkins P, Moriarty F, Ahmed I. Does premedication with mucolytic agents improve mucosal visualization during oesophagogastroduodenoscopy: a systematic review and meta-analysis. Surg Res Pract. 2021;2021:1570121. 10.1155/2021/1570121 PMC7846405 33553573

[48] Goto R, Arai K, Kitada H, Ogoshi K, Hamashima C. Labor resource use for endoscopic gastric cancer screening in Japanese primary care settings: a work sampling study. PLoS ONE. 2014;9:e88113. 10.1371/journal.pone.0088113 PMC3921128 24523875

[49] Kim SY, Park JM, Cho HS, Cho YK, Choi MG. Assessment of cimetropium bromide use for the detection of gastric neoplasms during esophagogastroduodenoscopy. JAMA Netw Open. 2022;5:e223827. 10.1001/jamanetworkopen.2022.3827 PMC8943631 35319761

[50] Iwagami H, Seta T, Nakano S, Wakita M, Konishi T, Matsumoto H, et al. Association between antispasmodics and detection of lesions by screening esophagogastroduodenoscopy. JGH Open. 2022;6:792–8. 10.1002/jgh3.12828 PMC9667398 36406644

[51] Wu Z, Li S, Lu L, Zhang Z, Wang G, Qin T, et al. Use of pronase in screening for early cancers of the upper gastrointestinal tract. Hong Kong Med J. 2025;31:41–7. 10.12809/hkmj2411578 39995335

[52] Li J, Wang L, Hu W, Wu J, Chen H, Wang L, et al. Effect of premedication with pronase before upper gastrointestinal endoscopy: a multicenter prospective randomized controlled study. J Clin Gastroenterol. 2024;58:53–6. 10.1097/MCG.0000000000001816 36507929

[53] Cha JM, Won KY, Chung IK, Kim GH, Lee SY, Cho YK. Effect of pronase premedication on narrow-band imaging endoscopy in patients with precancerous conditions of stomach. Dig Dis Sci. 2014;59:2735–41. 10.1007/s10620-014-3218-z 24861034

[54] Quach DT, Ho QD, Vu KV, Vu KT, Tran HV, Le NQ, et al. Improving efficacy of endoscopic diagnosis of early gastric cancer: gaps to overcome from the real-world practice in vietnam. Biomed Res Int. 2020;2020:7239075. 10.1155/2020/7239075 PMC7201490 32420364

[55] Sidhu R, Turnbull D, Haboubi H, Leeds JS, Healey C, Hebbar S, et al. British Society of Gastroenterology guidelines on sedation in gastrointestinal endoscopy. Gut. 2024;73:219–45. 10.1136/gutjnl-2023-330396 PMC10850688 37816587

[56] Gotoda T, Akamatsu T, Abe S, Shimatani M, Nakai Y, Hatta W, et al. Guidelines for sedation in gastroenterological endoscopy (second edition). Dig Endosc. 2021;33:21–53. 10.1111/den.13882 33124106

[57] Early DS, Lightdale JR, Vargo JJ 2nd, Acosta RD, Chandrasekhara V, Chathadi KV, et al. Guidelines for sedation and anesthesia in GI endoscopy. Gastrointest Endosc. 2018;87:327–37. 10.1016/j.gie.2017.07.018 29306520

[58] Fernández-Esparrach G, Marín-Gabriel JC, Díez Redondo P, Núñez H, Rodríguez de Santiago E, Rosón P, et al. Quality in diagnostic upper gastrointestinal endoscopy for the detection and surveillance of gastric cancer precursor lesions: position paper of AEG, SEED and SEAP. Gastroenterol Hepatol. 2021;44:448–64. 10.1016/j.gastrohep.2021.01.002 33609597

[59] Zhang Z, Gao N, Liu K, Ni M, Zhang X, Yan P, et al. Risk factors of missed early gastric cancer in endoscopic resected population: a retrospective, case-control study. Surg Endosc. 2024;38:4380–9. 10.1007/s00464-024-10970-0 38886230

[60] Wu H, Xin L, Lin JH, Wang QH, Li B, Jin ZD, et al. Association between sedation and small neoplasm detection during diagnostic esophagogastroduodenoscopy: a propensity score-matched retrospective study. Scand J Gastroenterol. 2022;57:603–9. 10.1080/00365521.2021.2023212 34983263

[61] Salvador I, Arau B, Andújar X, Ferrer C, Zabana Y, Ruiz L, et al. Intravenous sedation during esophagogastroduodenoscopy is associated with a reduced risk of missed gastric cancer. BMC Gastroenterol. 2025;25:377. 10.1186/s12876-025-03936-6 PMC12083126 40375118

[62] Kawamura T, Wada H, Sakiyama N, Ueda Y, Shirakawa A, Okada Y, et al. Examination time as a quality indicator of screening upper gastrointestinal endoscopy for asymptomatic examinees. Dig Endosc. 2017;29:569–75. 10.1111/den.12804 28066945

[63] Teh JL, Tan JR, Lau LJ, Saxena N, Salim A, Tay A, et al. Longer examination time improves detection of gastric cancer during diagnostic upper gastrointestinal endoscopy. Clin Gastroenterol Hepatol. 2015;13:480-7.e2. 10.1016/j.cgh.2014.07.059 25117772

[64] Park JM, Huo SM, Lee HH, Lee BI, Song HJ, Choi MG. Longer observation time increases proportion of neoplasms detected by esophagogastroduodenoscopy. Gastroenterology. 2017;153:460-9.e1. 10.1053/j.gastro.2017.05.009 28501581

[65] Dong L, Zhang X, Xuan Y, Xiong P, Ning Y, Zhang B, et al. Relationship between observation time and detection rate of focal lesions in esophagogastroduodenoscopy: a single-center, retrospective study. BMC Gastroenterol. 2024;24:75. 10.1186/s12876-024-03157-3 PMC10870502 38360548

[66] Lee HH, Park JM, Lim CH, Kim JS, Cho YK, Choi MG. The impact of pre-resection endoscopic examination time on the rate of synchronous gastric neoplasms missed during endoscopic treatment. Surg Endosc. 2017;31:3952–60. 10.1007/s00464-017-5428-x 28271265

[67] Yao K. The endoscopic diagnosis of early gastric cancer. Ann Gastroenterol. 2013;26:11–22. PMC3959505 24714327

[68] Lee JI, Kim JS, Kim B-W, Huh CW. Taking more gastroscopy images increases the detection rate of clinically significant gastric lesions: validation of a systematic screening protocol for the stomach. Korean J Helicobacter Up Gastrointest Res. 2020;20:225–32.

[69] Kashiwagi K, Yoshida T, Fukuhara K, Bessho R, Ichikawa H, Inoue N, et al. Optimal number of images and 2-year interval affect cancer detection in screening esophagogastroduodenoscopy: an observational study. Medicine (Baltimore). 2024;103:e38774. 10.1097/MD.0000000000038774 PMC11466123 38941380

[70] Rey JF, Lambert R. ESGE recommendations for quality control in gastrointestinal endoscopy: guidelines for image documentation in upper and lower GI endoscopy. Endoscopy. 2001;33:901–3. 10.1055/s-2001-42537 11605605

[71] Bisschops R, Areia M, Coron E, Dobru D, Kaskas B, Kuvaev R, et al. Performance measures for upper gastrointestinal endoscopy: a European Society of Gastrointestinal Endoscopy (ESGE) Quality Improvement Initiative. Endoscopy. 2016;48:843–64. 10.1055/s-0042-113128 27548885

[72] Ang TL, Pittayanon R, Lau JY, Rerknimitr R, Ho SH, Singh R, et al. A multicenter randomized comparison between high-definition white light endoscopy and narrow band imaging for detection of gastric lesions. Eur J Gastroenterol Hepatol. 2015;27:1473–8. 10.1097/MEG.0000000000000478 26426836

[73] Yoshida N, Doyama H, Yano T, Horimatsu T, Uedo N, Yamamoto Y, et al. Early gastric cancer detection in high-risk patients: a multicentre randomised controlled trial on the effect of second-generation narrow band imaging. Gut. 2021;70:67–75. 10.1136/gutjnl-2019-319631 PMC7788198 32241898

[74] Dohi O, Yagi N, Naito Y, Fukui A, Gen Y, Iwai N, et al. Blue laser imaging-bright improves the real-time detection rate of early gastric cancer: a randomized controlled study. Gastrointest Endosc. 2019;89:47–57. 10.1016/j.gie.2018.08.049 30189197

[75] Kadota T, Abe S, Uedo N, Doyama H, Furue Y, Muto M, et al. Comparison of effective imaging modalities for detecting gastric neoplasms: a randomized 3-arm phase II trial. Am J Gastroenterol. 2024;119:2010–8. 10.14309/ajg.0000000000002871 PMC11446510 38752623

[76] Manti M, Gkolfakis P, Kamperidis N, Toskas A, Papaefthymiou A, Tziatzios G, et al. Narrow-band imaging for the detection of early gastric cancer among high-risk patients: a systematic review and meta-analysis. Medicina (Kaunas). 2025. 10.3390/medicina61091613. PMC12471627 41011004

[77] Gao J, Zhang X, Meng Q, Jin H, Zhu Z, Wang Z, et al. Linked color imaging can improve detection rate of early gastric cancer in a high-risk population: a multi-center randomized controlled clinical trial. Dig Dis Sci. 2021;66:1212–9. 10.1007/s10620-020-06289-0 32363529

[78] Ono S, Kawada K, Dohi O, Kitamura S, Koike T, Hori S, et al. Linked color imaging focused on neoplasm detection in the upper gastrointestinal tract : a randomized trial. Ann Intern Med. 2021;174:18–24. 10.7326/M19-2561 33076693

[79] Min M, Sun X, Bai J, Zhang Q, Yang X, Guo Q, et al. Diagnostic accuracy of linked colour imaging versus white light imaging for early gastric cancers: a prospective, multicentre, randomized controlled trial study. Ann Med. 2022;54:3306–14. 10.1080/07853890.2022.2147991 PMC9704855 36411585

[80] Duan H, Zhou X, Li Q, Liu L, Wang Q, Wu K, et al. Diagnostic accuracy of linked color imaging and white light imaging for early gastric cancer and gastrointestinal metaplasia: a systematic review and meta-analysis. Front Oncol. 2024;14:1480651. 10.3389/fonc.2024.1480651 PMC11604575 39619434

[81] An W, Wu Q, Su XJ, Sun HX, Wang J, Dou WJ, et al. Optical enhancement mode 2 improves the detection rate of gastric neoplastic lesion in high-risk populations: a multicenter randomized controlled clinical study. United Eur Gastroenterol J. 2024;12:772–9. 10.1002/ueg2.12577 PMC11250407 38753528

[82] Kubo M, Ono S, Dohi O, Fukui H, Hikichi T, Kato T, et al. Surveillance esophagogastroduodenoscopy using linked color imaging and narrow-band imaging: a multicenter randomized controlled trial. J Gastroenterol Hepatol. 2024;39:1065–72. 10.1111/jgh.16511 38403475

[83] Rugge M, Meggio A, Pennelli G, Piscioli F, Giacomelli L, De Pretis G, et al. Gastritis staging in clinical practice: the OLGA staging system. Gut. 2007;56:631–6. 10.1136/gut.2006.106666 PMC1942143 17142647

[84] Capelle LG, de Vries AC, Haringsma J, Ter Borg F, de Vries RA, Bruno MJ, et al. The staging of gastritis with the OLGA system by using intestinal metaplasia as an accurate alternative for atrophic gastritis. Gastrointest Endosc. 2010;71:1150–8. 10.1016/j.gie.2009.12.029 20381801

[85] Dixon MF, Genta RM, Yardley JH, Correa P. Classification and grading of gastritis. the updated Sydney system. International workshop on the histopathology of gastritis, Houston 1994. Am J Surg Pathol. 1996;20:1161–81. 10.1097/00000478-199610000-00001 8827022

[86] Delgado Guillena PG, Cuatrecasas M, Montori S, Felipez Varela N, Llach J, Archilla I, et al. The OLGIMA system for gastric cancer risk assessment. a useful method based on the histological Sydney consensus. Histopathology. 2026;88:911–21. 10.1111/his.70042 PMC12891928 41293900

[87] Yue H, Shan L, Bin L. The significance of OLGA and OLGIM staging systems in the risk assessment of gastric cancer: a systematic review and meta-analysis. Gastric Cancer. 2018;21:579–87. 10.1007/s10120-018-0812-3 29460004

[88] Liu Y, Uemura N, Xiao SD, Tytgat GN, Kate FJ. Agreement between endoscopic and histological gastric atrophy scores. J Gastroenterol. 2005;40:123–7. 10.1007/s00535-004-1511-x 15770394

[89] Kono S, Gotoda T, Yoshida S, Oda I, Kondo H, Gatta L, et al. Can endoscopic atrophy predict histological atrophy? Historical study in United Kingdom and Japan. World J Gastroenterol. 2015;21:13113–23. 10.3748/wjg.v21.i46.13113 PMC4674730 26673849

[90] Quach DT, Le HM, Nguyen OT, Nguyen TS, Uemura N. The severity of endoscopic gastric atrophy could help to predict operative link on gastritis assessment gastritis stage. J Gastroenterol Hepatol. 2011;26:281–5. 10.1111/j.1440-1746.2010.06474.x 21261717

[91] Lim JH, Kim N, Lee HS, Choe G, Jo SY, Chon I, et al. Correlation between endoscopic and histological diagnoses of gastric intestinal metaplasia. Gut Liver. 2013;7:41–50. 10.5009/gnl.2013.7.1.41 PMC3572319 23423616

[92] Panteris V, Nikolopoulou S, Lountou A, Triantafillidis JK. Diagnostic capabilities of high-definition white light endoscopy for the diagnosis of gastric intestinal metaplasia and correlation with histologic and clinical data. Eur J Gastroenterol Hepatol. 2014;26:594–601. 10.1097/MEG.0000000000000097 24743505

[93] Esposito G, Pimentel-Nunes P, Angeletti S, Castro R, Libânio D, Galli G, et al. Endoscopic grading of gastric intestinal metaplasia (EGGIM): a multicenter validation study. Endoscopy. 2019;51:515–21. 10.1055/a-0808-3186 30577062

[94] Zhang G, Zheng J, Zheng L, Yu S, Jiang C, Lin W, et al. Gastric intestinal metaplasia assessment between linked color imaging based on endoscopy and pathology. Scand J Gastroenterol. 2021;56:103–10. 10.1080/00365521.2020.1849385 33232631

[95] Dinis-Ribeiro M, Libânio D, Uchima H, Spaander MCW, Bornschein J, Matysiak-Budnik T, et al. Management of epithelial precancerous conditions and early neoplasia of the stomach (MAPS III): European Society of Gastrointestinal Endoscopy (ESGE), European Helicobacter and Microbiota Study Group (EHMSG) and European Society of Pathology (ESP) guideline update 2025. Endoscopy. 2025;57:504–54. 10.1055/a-2529-5025 40112834

[96] Wu L, Zhang J, Zhou W, An P, Shen L, Liu J, et al. Randomised controlled trial of WISENSE, a real-time quality improving system for monitoring blind spots during esophagogastroduodenoscopy. Gut. 2019;68:2161–9. 10.1136/gutjnl-2018-317366 PMC6872441 30858305

[97] Wu L, He X, Liu M, Xie H, An P, Zhang J, et al. Evaluation of the effects of an artificial intelligence system on endoscopy quality and preliminary testing of its performance in detecting early gastric cancer: a randomized controlled trial. Endoscopy. 2021;53:1199–207. 10.1055/a-1350-5583 33429441

[98] Li YD, Li HZ, Chen SS, Jin CH, Chen M, Cheng M, et al. Correlation of the detection rate of upper GI cancer with artificial intelligence score: results from a multicenter trial (with video). Gastrointest Endosc. 2022;95:1138-46.e2. 10.1016/j.gie.2021.12.019 34973966

[99] Chan SM, Chan D, Yip HC, Scheppach MW, Lam R, Ng SK, et al. Artificial intelligence-assisted esophagogastroduodenoscopy improves procedure quality for endoscopists in early stages of training. Endosc Int Open. 2025;13:a25476645. 10.1055/a-2547-6645 PMC12042994 40309064

[100] Wu L, Shang R, Sharma P, Zhou W, Liu J, Yao L, et al. Effect of a deep learning-based system on the miss rate of gastric neoplasms during upper gastrointestinal endoscopy: a single-centre, tandem, randomised controlled trial. Lancet Gastroenterol Hepatol. 2021;6:700–8. 10.1016/S2468-1253(21)00216-8 34297944

[101] Zhou R, Liu J, Zhang C, Zhao Y, Su J, Niu Q, et al. Efficacy of a real-time intelligent quality-control system for the detection of early upper gastrointestinal neoplasms: a multicentre, single-blinded, randomised controlled trial. EClinicalMedicine. 2024;75:102803. 10.1016/j.eclinm.2024.102803 PMC11402435 39281103

[102] Gong EJ, Bang CS, Lee JJ, Baik GH, Lim H, Jeong JH, et al. Deep learning-based clinical decision support system for gastric neoplasms in real-time endoscopy: development and validation study. Endoscopy. 2023;55:701–8. 10.1055/a-2031-0691 36754065

[103] Huang L, Xu M, Li Y, Dong Z, Lin J, Wang W, et al. Gastric neoplasm detection of computer-aided detection-assisted esophagogastroduodenoscopy changes with implement scenarios: a real-world study. J Gastroenterol Hepatol. 2024;39:2787–95. 10.1111/jgh.16784 39469909

[104] Adachi K, Ebisutani Y, Matsubara Y, Okimoto E, Ishimura N, Ishihara S. Effectiveness of artificial intelligence in screening esophagogastroduodenoscopy. Cureus. 2025;17:e79935. 10.7759/cureus.79935 PMC11962169 40177429

[105] Xu Z, Li Y, Su P, Zhong Z, Zeng Z, Chen M, et al. Artificial intelligence system improves the quality of digestive endoscopy: a prospective pretest and post-test single-center clinical trial. Dig Liver Dis. 2025;57:1830–7. 10.1016/j.dld.2025.04.029 40345942

