# Supplementary material for: Screening strategies for the early detection of non-cardia gastric cancer in low- to moderate-prevalence regions: Asia-Pacific gastroesophageal cancer congress consensus 2026
Source: Gastric Cancer. 2026 Oct 14;29(6):1185–205. doi: 10.1007/s10120-026-01800-4 (PMC13754983; doi:10.1007/s10120-026-01800-4)
Supplement: Supplementary file 1 [file 10120_2026_1800_MOESM1_ESM.docx]

**Supplementary Table 1. PubMed search strategies and search results for each clinical question**

| **CQ** | **PubMed search strategy** | **Records identified, n** |
| --- | --- | --- |
| CQ1 | (gastric[Title/Abstract] OR stomach[Title/Abstract]) AND (cancer[Title/Abstract] OR precancerous[Title/Abstract] OR malignant[Title/Abstract] OR neoplasm[Title/Abstract] OR neoplasms[Title/Abstract] OR dysplasia[Title/Abstract]) AND (early[Title/Abstract] OR early-stage[Title/Abstract] OR localized[Title/Abstract] OR localized-stage[Title/Abstract]) AND (screening[Title/Abstract] OR screen[Title/Abstract] OR detection[Title/Abstract] OR diagnosis[Title/Abstract] OR diagnosing[Title/Abstract] OR detect[Title/Abstract] OR identifying[Title/Abstract] OR identify[Title/Abstract] OR identification[Title/Abstract]) AND (endoscopy[Title/Abstract] OR esophagogastroduodenoscopy[Title/Abstract] OR EGD[Title/Abstract] OR gastroscopy[Title/Abstract]) | 2,040 |
| CQ2 | (gastric[Title/Abstract] OR stomach[Title/Abstract]) AND (cancer[Title/Abstract] OR precancerous[Title/Abstract] OR malignant[Title/Abstract] OR neoplasm[Title/Abstract] OR neoplasms[Title/Abstract] OR dysplasia[Title/Abstract]) AND (early[Title/Abstract] OR early-stage[Title/Abstract] OR localized[Title/Abstract] OR localized-stage[Title/Abstract]) AND (screening[Title/Abstract] OR screen[Title/Abstract] OR detection[Title/Abstract] OR diagnosis[Title/Abstract] OR diagnosing[Title/Abstract] OR detect[Title/Abstract] OR identifying[Title/Abstract] OR identify[Title/Abstract] OR identification[Title/Abstract]) AND (endoscopy[Title/Abstract] OR esophagogastroduodenoscopy[Title/Abstract] OR EGD[Title/Abstract] OR gastroscopy[Title/Abstract]) AND ("upper gastrointestinal series"[Title/Abstract] OR UGI[Title/Abstract] OR radiography[Title/Abstract] OR X-ray[Title/Abstract] OR barium[Title/Abstract]) | 150 |
| CQ3 | ((gastric[Title/Abstract] OR stomach[Title/Abstract]) AND (cancer[Title/Abstract] OR precancerous[Title/Abstract] OR malignant[Title/Abstract] OR neoplasm[Title/Abstract] OR neoplasms[Title/Abstract] OR dysplasia[Title/Abstract]) AND (early[Title/Abstract] OR early-stage[Title/Abstract])) AND (screening[Title/Abstract] OR screen[Title/Abstract] OR detection[Title/Abstract] OR diagnosis[Title/Abstract] OR diagnosing[Title/Abstract] OR detect[Title/Abstract] OR identifying[Title/Abstract] OR identify[Title/Abstract] OR identification[Title/Abstract]) AND (risk stratification[Title/Abstract] OR risk prediction[Title/Abstract] OR risk assessment[Title/Abstract]) | 195 |
| CQ4 | (gastric[Title/Abstract] OR stomach[Title/Abstract]) AND (cancer[Title/Abstract] OR precancerous[Title/Abstract] OR malignant[Title/Abstract] OR neoplasm[Title/Abstract] OR neoplasms[Title/Abstract] OR dysplasia[Title/Abstract]) AND (early[Title/Abstract] OR early-stage[Title/Abstract]) AND (screening[Title/Abstract] OR screen[Title/Abstract] OR detection[Title/Abstract] OR diagnosis[Title/Abstract] OR diagnosing[Title/Abstract] OR detect[Title/Abstract] OR identifying[Title/Abstract] OR identify[Title/Abstract] OR identification[Title/Abstract]) AND (serological[Title/Abstract] OR serum[Title/Abstract] OR blood[Title/Abstract]) AND (biomarker[Title/Abstract] OR marker[Title/Abstract]) | 479 |
| CQ5 | (gastric[Title/Abstract] OR stomach[Title/Abstract]) AND (cancer[Title/Abstract] OR precancerous[Title/Abstract] OR malignant[Title/Abstract] OR neoplasm[Title/Abstract] OR neoplasms[Title/Abstract] OR dysplasia[Title/Abstract]) AND (screening[Title/Abstract] OR screen[Title/Abstract] OR detection[Title/Abstract] OR diagnosis[Title/Abstract] OR diagnosing[Title/Abstract] OR detect[Title/Abstract] OR identifying[Title/Abstract] OR identify[Title/Abstract] OR identification[Title/Abstract] OR "miss rate"[Title/Abstract] OR "missing cancer"[Title/Abstract] OR visibility[Title/Abstract] OR quality[Title/Abstract]) AND ("oral preparation"[Title/Abstract] OR simethicone[Title/Abstract] OR "N-acetylcysteine"[Title/Abstract] OR "mucolytic agent"[Title/Abstract] OR buscopan[Title/Abstract] OR Hyoscine-N-butylbromide[Title/Abstract] OR Butylscopolamine[Title/Abstract] OR "scopolamine butylbromide"[Title/Abstract] OR antispasmodic[Title/Abstract] OR premedication[Title/Abstract] OR "pre-endoscopy preparation"[Title/Abstract] OR pronase[Title/Abstract] OR protease[Title/Abstract]) | 154 |
| CQ6 | (gastric[Title/Abstract] OR stomach[Title/Abstract]) AND (cancer[Title/Abstract] OR precancerous[Title/Abstract] OR malignant[Title/Abstract] OR neoplasm[Title/Abstract] OR neoplasms[Title/Abstract] OR dysplasia[Title/Abstract]) AND (screening[Title/Abstract] OR screen[Title/Abstract] OR detection[Title/Abstract] OR diagnosis[Title/Abstract] OR diagnosing[Title/Abstract] OR detect[Title/Abstract] OR identifying[Title/Abstract] OR identify[Title/Abstract] OR identification[Title/Abstract] OR "miss rate"[Title/Abstract] OR "missing cancer"[Title/Abstract]) AND (sedation[Title/Abstract] OR "conscious sedation"[Title/Abstract] OR "deep sedation"[Title/Abstract] OR sedative[Title/Abstract] OR sedatives[Title/Abstract] OR midazolam[Title/Abstract] OR propofol[Title/Abstract] OR etomidate[Title/Abstract] OR dexmedetomidine[Title/Abstract] OR flunitrazepam[Title/Abstract] OR diazepam[Title/Abstract] OR remimazolam[Title/Abstract] OR fentanyl[Title/Abstract] OR pethidine[Title/Abstract]) | 69 |
| CQ7 | (gastric[Title/Abstract] OR stomach[Title/Abstract]) AND (cancer[Title/Abstract] OR precancerous[Title/Abstract] OR malignant[Title/Abstract] OR neoplasm[Title/Abstract] OR neoplasms[Title/Abstract] OR dysplasia[Title/Abstract]) AND (screening[Title/Abstract] OR screen[Title/Abstract] OR detection[Title/Abstract] OR diagnosis[Title/Abstract] OR diagnosing[Title/Abstract] OR detect[Title/Abstract] OR identifying[Title/Abstract] OR identify[Title/Abstract] OR identification[Title/Abstract]) AND (observation[Title] OR examination[Title]) | 210 |
| CQ8 | (gastric[Title/Abstract] OR stomach[Title/Abstract]) AND (cancer[Title/Abstract] OR precancerous[Title/Abstract] OR malignant[Title/Abstract] OR neoplasm[Title/Abstract] OR neoplasms[Title/Abstract] OR dysplasia[Title/Abstract]) AND (screening[Title/Abstract] OR screen[Title/Abstract] OR detection[Title/Abstract] OR diagnosis[Title/Abstract] OR diagnosing[Title/Abstract] OR detect[Title/Abstract] OR identifying[Title/Abstract] OR identify[Title/Abstract] OR identification[Title/Abstract]) AND (image[Title/Abstract] OR photo[Title/Abstract] OR picture[Title/Abstract] OR photograph[Title/Abstract]) NOT (review[Publication Type]) | 771 |
| CQ9 | (gastric[Title/Abstract] OR stomach[Title/Abstract]) AND (cancer[Title/Abstract] OR malignant[Title/Abstract] OR neoplasm[Title/Abstract] OR neoplasms[Title/Abstract] OR dysplasia[Title/Abstract]) AND (screening[Title/Abstract] OR screen[Title/Abstract] OR detection[Title/Abstract] OR diagnosis[Title/Abstract] OR diagnosing[Title/Abstract] OR detect[Title/Abstract] OR identifying[Title/Abstract] OR identify[Title/Abstract] OR identification[Title/Abstract]) AND ("image-enhanced endoscopy"[Title/Abstract] OR "image enhancement"[Title/Abstract] OR "narrow-band imaging"[Title/Abstract] OR NBI[Title/Abstract] OR "texture and color enhancement"[Title/Abstract] OR TXI[Title/Abstract] OR "blue laser imaging"[Title/Abstract] OR BLI[Title/Abstract] OR "linked color imaging"[Title/Abstract] OR LCI[Title/Abstract] OR "optical enhancement"[Title/Abstract] OR OE[Title/Abstract]) | 555 |
| CQ10 | (gastric[Title/Abstract] OR stomach[Title/Abstract]) AND (atrophy[Title/Abstract] OR "intestinal metaplasia"[Title/Abstract] OR IM[Title/Abstract] OR GIM[Title/Abstract]) AND ("image-enhanced endoscopy"[Title/Abstract] OR "narrow-band imaging"[Title/Abstract] OR NBI[Title/Abstract] OR "texture and color enhancement"[Title/Abstract] OR TXI[Title/Abstract] OR "linked color imaging"[Title/Abstract] OR LCI[Title/Abstract] OR "blue laser imaging"[Title/Abstract] OR BLI[Title/Abstract] OR "optical enhancement"[Title/Abstract] OR OE[Title/Abstract] OR chromoendoscopy[Title/Abstract]) AND (diagnosis[Title/Abstract] OR assessment[Title/Abstract] OR accuracy[Title/Abstract] OR sensitivity[Title/Abstract] OR specificity[Title/Abstract] OR EGGIM[Title/Abstract]) | 214 |
| CQ11 | (gastric[Title/Abstract] OR stomach[Title/Abstract]) AND (cancer[Title/Abstract] OR precancerous[Title/Abstract] OR malignant[Title/Abstract] OR neoplasm[Title/Abstract] OR neoplasms[Title/Abstract] OR dysplasia[Title/Abstract]) AND (screening[Title/Abstract] OR screen[Title/Abstract] OR detection[Title/Abstract] OR diagnosis[Title/Abstract] OR diagnosing[Title/Abstract] OR detect[Title/Abstract] OR identifying[Title/Abstract] OR identify[Title/Abstract] OR identification[Title/Abstract]) AND ("artificial intelligence"[Title/Abstract] OR "deep-learning"[Title/Abstract] OR "neural network"[Title/Abstract] OR "computer analysis"[Title/Abstract] OR "computer aided"[Title/Abstract]) | 699 |

Literature searches were performed in PubMed without date restrictions. The final search was conducted in July 2025. **The searches were rerun using the original search strategies with a publication date cutoff of July 31, 2025, to confirm the number of records identified.**

CQ, clinical question
